# Structural insights into the *Thermus thermophilus* type IV pilus machinery assembling two distinct pili

**DOI:** 10.1038/s42003-026-09762-0

**Published:** 2026-03-31

**Authors:** Alexander Neuhaus, Mathew McLaren, Michail N. Isupov, Matthew Gaines, Emma Buzzard, Mateusz Sikora, Cyril Hanus, Bertram Daum, Beate Averhoff, Vicki A. M. Gold

**Affiliations:** 1 Living Systems Institute, University of Exeter, Stocker Road, Exeter, UK; 2 Faculty of Health and Life Sciences, University of Exeter, Exeter, UK; 3 Department of Theoretical Biophysics, Max Planck Institute of Biophysics, Frankfurt am Main, Germany; 4 Malopolska Centre of Biotechnology, Jagiellonian University, Kraków, Poland; 5 Institute of Psychiatry and Neurosciences of Paris, Inserm UMR1266 - Université Paris Cité, Paris, France; 6 GHU Psychiatrie et Neurosciences de Paris, Paris, France; 7 Molecular Microbiology and Bioenergetics, Institute of Molecular Biosciences, Johann Wolfgang Goethe University, Frankfurt am Main, Germany; 8 Present Address: Institute for Medical Physics and Biophysics and Center for Soft Nanoscience, University of Münster, Münster, Germany; 9 Present Address: Department of Biochemistry, University of Wisconsin, Madison, WI USA; 10 Present Address: Wisconsin Energy Institute and Great Lakes Bioenergy Research Center, University of Wisconsin-Madison, Madison, WI USA; 11 Present Address: Electron Bio-Imaging Centre (eBIC), Diamond Light Source Ltd, Diamond House, Harwell Science and Innovation Campus, Didcot, UK

**Keywords:** Cryoelectron tomography, Cryoelectron microscopy, Protein structure predictions

## Abstract

Type IV pili are long, filamentous structures that extend from bacterial cell surfaces, enabling cells to respond to changing environments and facilitating genome plasticity. *Thermus thermophilus* HB27 produces two different type IV pili, each exhibiting distinct structural and functional properties. Here, we combine cryo-electron tomography, mutagenesis, and AlphaFold predictions to generate hypothetical in situ models of the *T. thermophilus* type IV pilus assembly machinery. Using single-particle cryo-electron microscopy, we determine structures of both filament types, enabling modelling of their surface glycans. Molecular dynamics simulations further reveal the flexibility of these glycans on extrusion. Integration of the filament structures with our hypothetical model of the assembly machinery offers a framework for further dissecting T4P architecture and biogenesis.

## Abstract (web summary)

Thermus thermophilus forms two distinct type IV pili. Cryo-electron microscopy, mutagenesis, structural predictions and simulations reveal their structures, glycan flexibility, and a hypothetical model of pilus assembly.

## Introduction

Type IV pili (T4P) are protein filaments assembled on the surface of many bacterial species. They play important roles in diverse functions, including surface motility and adhesion, and are linked to natural transformation systems that are critical for horizontal gene transfer. T4P have been extensively studied in a wide range of bacteria due to their importance in biofilm formation and pathogenesis^1^. They can act as mechanosensors, transmitting signals in pathogens that trigger the expression of virulence-associated genes^2^.

Across the bacterial kingdom, T4P assembly systems are structurally and functionally diverse, yet they share a conserved core machinery^3–5^. In Gram-negative bacteria, this machinery spans both outer and cytoplasmic membranes, and its core components include retractable filaments (T4P), a cytoplasmic membrane-embedded assembly platform, cytoplasmic motor proteins responsible for T4P assembly and retraction, and an outer membrane channel formed by a secretin family protein. *Thermus thermophilus* is a Gram-negative, thermophilic bacterium that, due to its efficient DNA uptake system^6,7^, has been used as a model system for studying T4P assembly and natural transformation for many years.

T4P are comprised of thousands of copies of pilin subunits, forming filaments a few nanometres in diameter but extending several micrometres in length. In our earlier work, we discovered that *T. thermophilus* assembles two distinct T4P - a wider and a narrower form^8^. Using single-particle cryo-electron microscopy (cryoEM), we determined the structures of both filaments to show that they differ in both protein composition and helical parameters. The wide pilus is composed of the pilin PilA4 (previously referred to as the “major” pilin), whereas the narrow pilus is composed of PilA5, which we identified in that study and found to be present at comparable abundance under optimal growth conditions^8^. We also biochemically identified glycan modifications decorating the surfaces of both filaments. However, due to limited resolution of the cryoEM maps, these glycans could not be modelled onto the structures.

The secretin responsible for extrusion of T4P across the outer membrane is called PilQ. Its reported stoichiometry varies between species, ranging from 12 to 15 copies^9–13^, and may differ within a single species, with both 14- and 15- fold assemblies observed^11^. In *Thermus*, the PilQ complex has been suggested to comprise 13 subunits, forming a double-gated channel embedded in the outer membrane via its C-terminal domain^13^. The N-terminal domains link PilQ with the cytoplasmic membrane assembly platform, comprised of the proteins PilM, PilN, PilO and PilC^14^. In the cytoplasm, motor proteins drive ATP-dependent cycles of pilus extension and retraction, with surface adhesion at the filament tip enabling T4P-mediated motility. In *T. thermophilus*, 3 T4P-associated ATPases have been identified and partially characterised: PilF, PilT1 and PilT2. PilF has been shown to play a role in T4P assembly, while PilT1/2 are associated with T4P disassembly^15^. It is possible that ATP hydrolysis results in conformational changes that lead to the rotation of PilC to add or remove T4P subunits^5,16^.

The *T. thermophilus* T4P machinery encodes an additional component, PilW, which lacks sequence similarity to known T4P machinery proteins from other species^17^. Interestingly, *pilW* is encoded in the equivalent genomic position (between *pilO* and *pilQ*) as *pilP* in other species^18^, where PilP is known to interact with the secretin PilQ^19^. Additionally, PilW contains a C-terminal domain which is structurally similar to the homology region (HR) of GspC from the type II secretion systems (T2SS), which is known to interact with secretin proteins^20^. Therefore, PilW may play a comparable function to PilP and the GspC family proteins by bridging PilQ to the cytoplasmic membrane assembly platform. This is consistent with the finding that PilW and PilQ form complexes with the cytoplasmic membrane assembly proteins PilM, PilN and PilO^21^, where PilW is required for correct PilQ assembly and/or stability^22^.

There are many unanswered questions regarding the function and organisation of the T4P machinery in *T. thermophilus*. For example, what is the structural arrangement of the individual components that comprise the machinery? What conformational changes occur during pilus assembly and retraction? How do the surface glycans contribute to pilus stability, flexibility, or interactions with the environment? How does a single machinery coordinate both twitching motility and natural transformation? To address these questions and guide further research, models are required to enable structure-guided mutagenesis and hypothesis-driven testing.

We had previously determined sub-tomogram average maps of the T4P machinery in situ in both piliated and non-piliated states using cryo-electron tomography (cryoET)^23^. Other research groups have also employed similar methods to reveal the molecular architecture of homologous systems^9,12,24^. Our earlier study predated both the development of AlphaFold for protein structure prediction and our discovery of two distinct T4P filaments. In this work, we build on that foundation by employing cryoET with a series of mutants to help identify proteins within the machinery. The mutants reveal considerable conformational flexibility, which limits the achievable resolution. Nevertheless, by integrating these findings with AlphaFold-based predictions, we can propose hypothetical models of non-piliated and the two piliated forms of the T4P machinery. We suggest that PilW acts as a flexible linker connecting the outer and cytoplasmic membrane components, enabling it to adjust to the upward shift of the cytoplasmic membrane assembly platform during pilus extrusion. In addition, using single-particle cryoEM and molecular dynamics simulations, we investigate T4P extrusion through the complex. This study offers a molecular framework for future, more detailed structure-function investigations of the T4P system.

## Results and discussion

### Spatial mapping of T4P machinery components

**Fig. 1:**
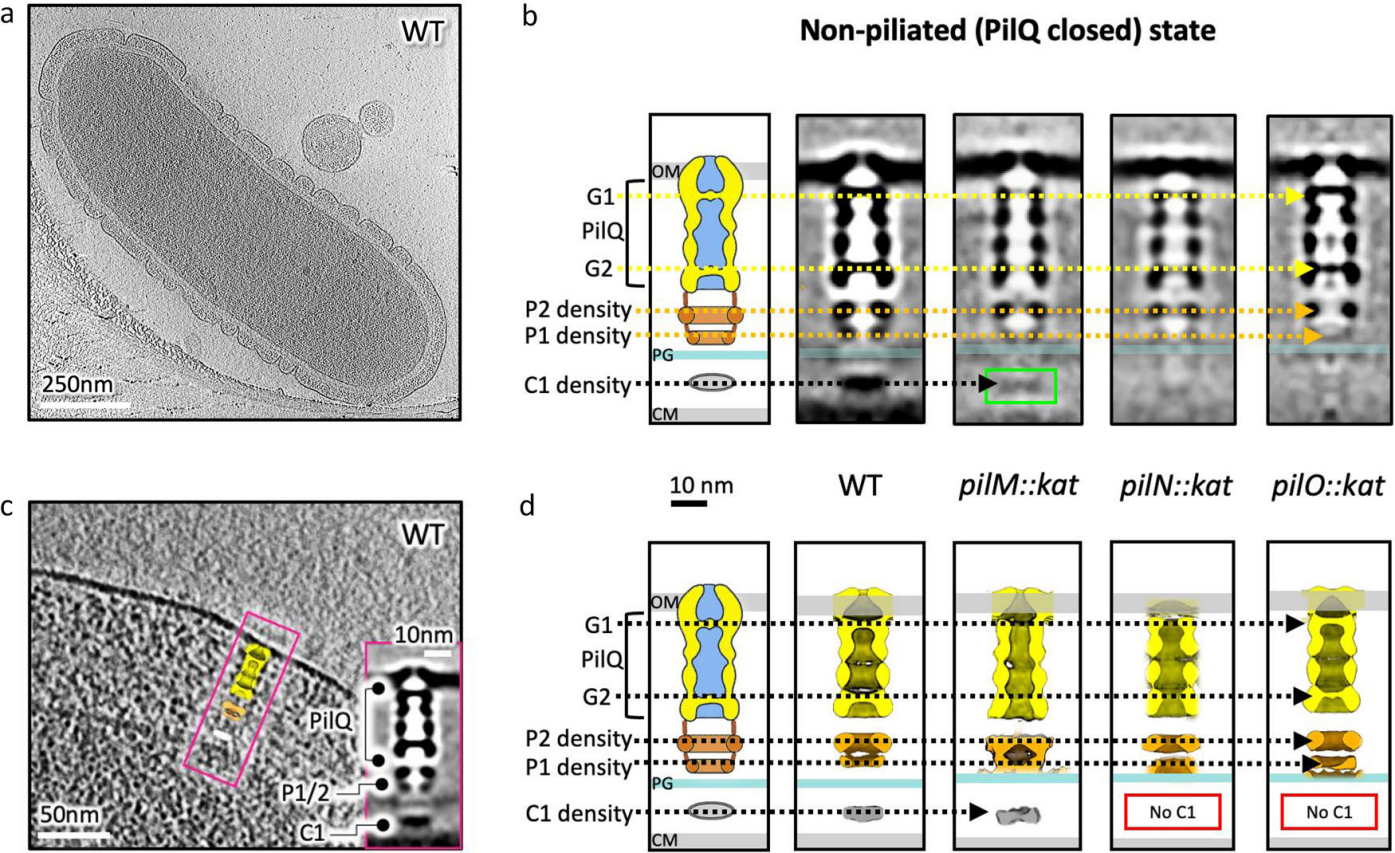
Non-piliated T4P machinery mutants. **a** Slice through a cryo tomogram of a wild-type (WT) *T. thermophilus* cell. Outer membrane invaginations of unknown function are present in all cells and do not correlate with the location of the T4P machinery. **b** A cartoon schematic of the non-piliated WT machinery alongside a slice through a sub-tomogram average of the WT (EMD-3021)^23^, with comparisons to mutant strains, aligned at the outer membrane. PilQ is shown in yellow, P1 and P2 densities in orange, and C1 density in grey, with dashed lines indicating corresponding positions across mutants. Gate 1 (G1) and Gate 2 (G2) of PilQ are indicated with yellow dashed lines. The green box highlights C1 density that is present, but less defined, in the *pilM::kat* mutant. OM, outer membrane; PG, peptidoglycan; CM, cytoplasmic membrane. **c** Enlarged view of a WT cell with a boxed inset showing the sub-tomogram average of the non-piliated machinery (EMD-3021)^23^, as well as the same volume mapped back into the tomogram. PilQ is shown in yellow, P1 and P2 densities in orange, and the C1 density in white. **d** Illustrative representations of the most prominent density in the sub-tomogram average maps for the complexes shown in (**b**). The C1 density in *pilM::kat* is weaker than that of the WT and becomes clearly visible only at lower contour levels. Overlays of the mutant complexes with the WT, displayed with proteins at the same contour level within each map, are shown in Supplementary Fig. 1. Red boxes indicate missing C1 density.

**Fig. 2:**
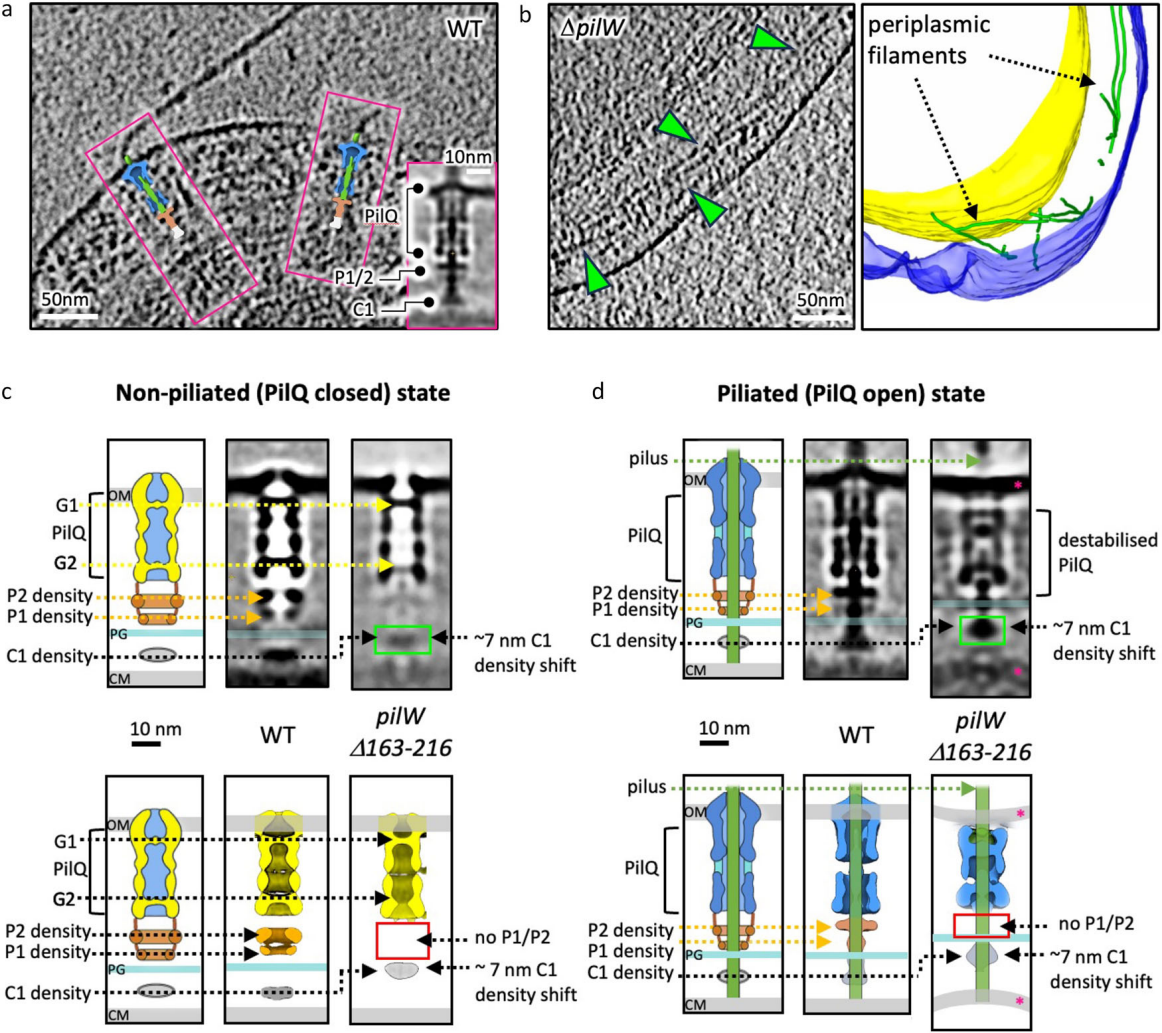
Effects of PilW mutation on the T4P machinery. **a** Enlarged view of a WT cell with a boxed inset showing the sub-tomogram average of the piliated machinery (EMD-3023)^23^, as well as the same volume mapped back into the tomogram. PilQ is shown in blue, P1 and P2 densities in orange, and the C1 density in white. **b** Slice through a cryo-tomogram of a Δ*pilW* cell with the corresponding 3D view. The outer membrane is shown in purple, the cytoplasmic membrane in yellow, and filaments trapped in the periplasm in green. **c** Upper panels: cartoon schematic with slices through sub-tomogram averages of non-piliated complexes from the *pilWΔ163-216* mutant compared to the WT (EMD-3021)^23^, aligned at the outer membrane. Lower panels: illustrative representation of the most prominent density in the sub-tomogram average maps. PilQ is shown in yellow, P1 and P2 densities in orange, and the C1 density in grey, with dashed lines indicating corresponding positions in the *pilWΔ163-216* mutant strain. Boxed regions highlight C1 density that has shifted ~7 nm towards PilQ in the *pilWΔ163-216* mutant. An overlay of non-piliated *pilWΔ163-216* mutant complex with the WT is shown in Supplementary Fig. 1. OM, outer membrane; PG, peptidoglycan; CM, cytoplasmic membrane. **d** Upper panels: Cartoon schematic with a slice through a sub-tomogram average of piliated complexes from the *pilWΔ163-216* mutant compared to the WT (EMD-3023)^23^, aligned at the outer membrane. Lower panels: illustrative 3D representations of the protein densities, coloured as per panel (**c**), additionally with the open state of PilQ (blue) and the pilus (green). Boxed regions highlight C1 density that has shifted ~7 nm towards PilQ. Pink asterisks indicate concave membranes. An overlay of the non-piliated *pilWΔ163-216* mutant complex with the WT is shown in Supplementary Fig. 1.

Imaging wild-type (WT) *T. thermophilus* bacteria (Fig. 1a) allows visualisation of T4P complexes spanning the periplasm in non-piliated and piliated states (Figs. 1c and 2a). In our earlier work, sub-tomogram averaging of these complexes revealed the position of the double-gated secretin PilQ, along with several unidentified periplasmic protein densities, which we grouped as P1, P2 and C1^23^ (boxed insets in Figs. 1c and 2a). To propose a hypothetical model of the T4P assembly machinery, these densities must be assigned to specific proteins. Therefore, we conducted sub-tomogram averaging of T4P complexes from mutant strains to assess changes in the overall organisation of the complex.

We first examined cells with a mutation in PilM, a protein expected to be cytosolic, linking to the membrane proteins PilN and PilO^25–27^. Piliated cells were not observed, indicating that PilM is essential for pilus assembly, consistent with biochemical data^14^. In the non-piliated state, particle abundance was reduced significantly compared to WT (Supplementary Table 1). Despite limited resolution, the periplasmic protein densities C1, P1 and P2 appear broadly similar to those in the WT (Fig. 1b, d, Supplementary Fig. 1a, c). These observations confirm that PilM does not constitute a major part of the periplasmic domains of the machinery. However, some protein densities are less well defined than in the WT, likely reflecting reduced complex stability due to the absence of PilM and/or a lower particle count (see Supplementary Note 1 “Impact of T4P assembly mutations on complex stability”).

Due to the proximity of the C1 density to the cytoplasmic membrane, we reasoned that the most likely protein candidates to localise here are the membrane proteins PilN and PilO. These homologous proteins form heterodimers^26,27^, and bind to a ring of cytoplasmic PilM proteins via the N-terminus of PilN^25^. In cells with PilN or PilO mutations, pili were again not observed. In these non-piliated forms, PilQ, P1 and P2 were visible; however, similar to the PilM mutant, densities were less well defined compared to the WT (Fig. 1b, d, Supplementary Fig. 1a, c), likely for similar reasons. Interestingly, in both PilN and PilO mutants, density for the cytoplasmic membrane-proximal C1 density is not visible at the size and position observed in the WT, consistent with both proteins containing transmembrane domains. These observations suggest that neither PilN nor PilO are major components of P1 or P2 and may correspond to the C1 density.

We reasoned that the only other candidate protein that could contribute to the P1 and P2 densities is PilW, which has been shown to associate with the outer membrane^17^, form high molecular weight heteropolymeric complexes with PilQ^22^, and link PilQ to the cytoplasmic membrane assembly platform^18^. We attempted to image the T4P machinery in cells lacking PilW but were unable to identify any T4P complexes. Interestingly, filaments were observed trapped in the periplasm (Fig. 2b). This is consistent with previous findings demonstrating the importance of PilW for the assembly and/or stability of PilQ^22^, which is required for T4P extrusion, and supports a role for PilW in linking the cytoplasmic membrane assembly platform to the secretin^21,22^. As the assembly platform is distinct and located in an opposing membrane to PilQ, we assume T4P are assembled and not directed properly to the secretin in the absence of PilW. Periplasmic pili have also been observed in *N. meningitidis* in the absence of PilQ and the retraction ATPase^28^. This indicates a common phenomenon where T4P become trapped in the periplasm because they cannot be extruded.

To localise PilW, we next imaged cells with a truncation of 54 residues in the protein (*pilWΔ163-216*) to determine whether a loss of mass would reveal changes in the sub-tomogram average maps. Interestingly, despite the truncation, the PilW mutant was able to assemble T4P, allowing us to compare the arrangement of proteins in both the non-piliated and piliated states with the WT (Fig. 2c, d, Supplementary Fig. 1a–c). In the mutant, the P1 and P2 densities beneath PilQ were not visible clearly (Fig. 2c, Supplementary Fig. 1a), whereas C1 was identified shifted ~7 nm towards PilQ in both non-piliated and piliated forms (Fig. 2c, d, Supplementary Fig. 1a, b).

Assembly of T4P in the PilW truncation mutant also affects the architecture of the outer and cytoplasmic membranes, which adopt a more concave curvature. This effect is most pronounced in the piliated state, where both membranes are observed drawn inward toward the periplasm surrounding the machinery (Fig. 2d). These observations support the idea that regions of PilW adopt a flexible, extended conformation^22^, allowing the protein to span variable periplasmic widths and facilitate alignment between components embedded in opposing membranes. PilW in *Thermus* is 292 amino acids long, ~60% longer than its proposed functional homologue PilP in *M. xanthus* and *P. aeruginosa*. This increased length likely reflects the broader periplasmic space in *Thermus* (~70 nm)^23,29^ compared to other Gram-negative bacteria (~30 nm)^9,12^. Our model is therefore consistent with previous models suggesting that PilP localises to the periplasm in *P. aeruginosa*^9^ and *M. xanthus*^12^. The comparatively high glycine and proline content in PilW likely contributes to its structural flexibility by disrupting regular secondary structure and favouring extended or disordered conformations^30^.

These results suggest that PilW likely constitutes or fully accounts for the P1 and P2 densities, linking PilQ with the PilN-PilO complex, which in turn interacts with PilM in the cytosol. To evaluate whether this proposed arrangement is consistent with the sub-tomogram average maps, we docked a combination of experimental and AlphaFold predicted models into sub-tomogram averages.

### Proposed architecture of the T4P machinery

**Fig. 3:**
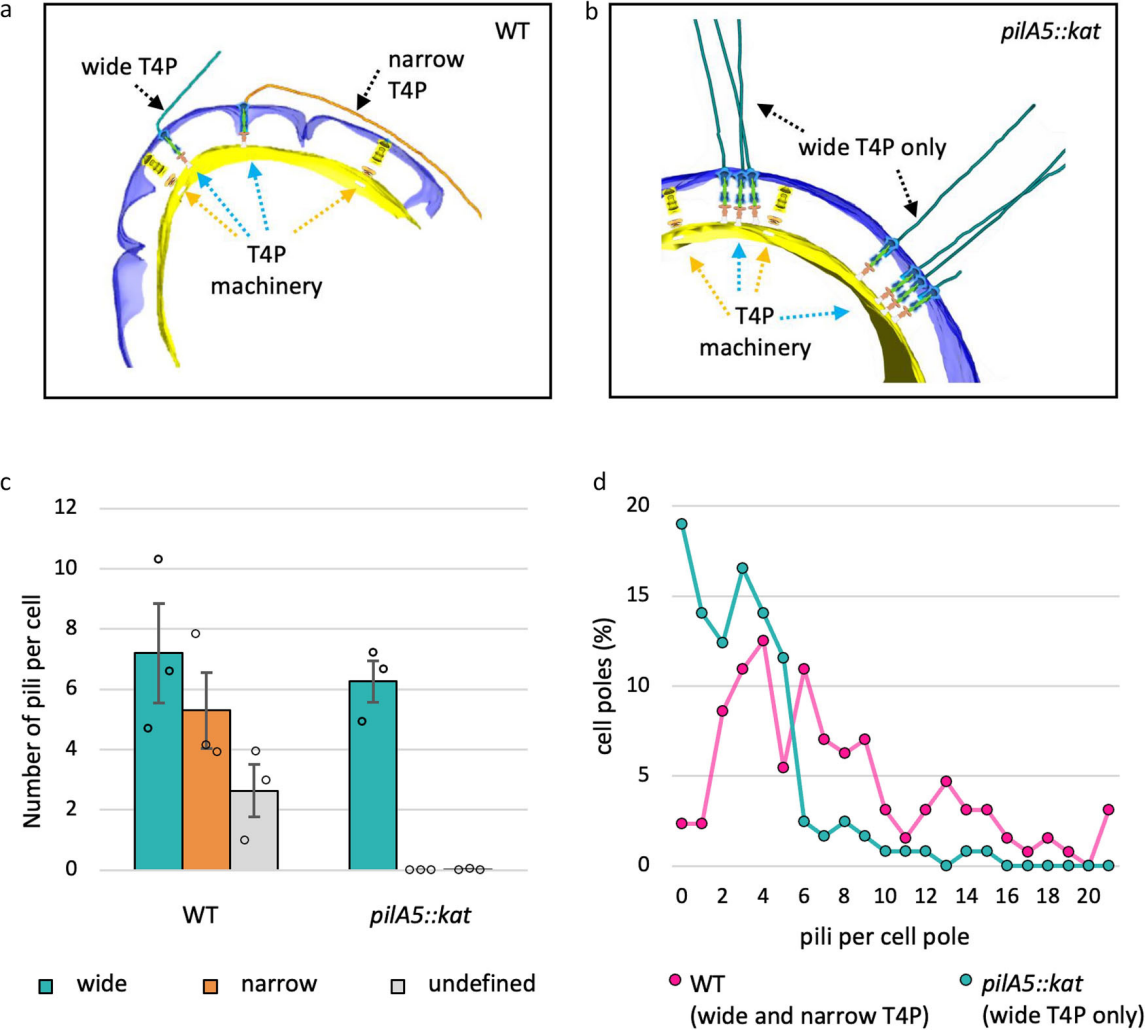
Loss of wide pili is not compensated by an increase in the narrow form. 3D views of cell poles with assembled T4P in (**a**) WT and (**b**) *pilA5::kat* mutant cells. Blue arrows indicate piliated T4P complexes and orange arrows indicate non-piliated complexes. The outer membrane is shown in purple and the cytoplasmic membrane in yellow. **c** Quantification of wide and narrow pili assembled on wild-type and *pilA5:kat* mutant cells. Values are presented as mean +/− standard error of the mean. *n* = 3 biological replicates per strain, with a minimum of 35 cell poles counted per replicate. These data were previously part of a larger data set in Supplementary Fig. 3c of our earlier work^8^ and are presented here to corroborate the imaging data in (**a**, **b**). The article was licensed under a Creative Commons Attribution 4.0 International License, which permits use, sharing, adaptation, distribution and reproduction in any medium or format (https://creativecommons.org/licenses/by/4.0/). **d** Scatter plot showing the percentage of cell poles expressing 0–20 pili. PilA4 (wide T4P) only expressing cells in the *pilA5::kat* mutant are shown in teal, and WT cells (wide and narrow T4P) in pink.

To avoid mixed populations of T4P machineries assembling both wide and narrow pili, we used a mutant cell line that exclusively assembles wide filaments comprised of PilA4^8^. This strain carries a mutation in the *pilA5* gene, which encodes the protein responsible for forming narrow pili. Previously, we developed an approach for accurately and objectively quantifying surface-exposed filaments, using electron microscopy and 2D classification to group T4P into different classes^8^. We confirmed that the WT strain produces both wide and narrow T4P whereas the *pilA5* mutant used for sub-tomogram averaging expresses only the wide (Fig. 3a, b, Supplementary Figs. 2, 3)^8^. Quantification of T4P showed that both WT and mutant strains assemble a comparable number of wide filaments (Fig. 3c). Notably, ~23% of WT cells exhibited hyperpiliation (defined as >10 T4P per pole), compared to only ~3% in *pilA5* mutant (Fig. 3d). In contrast, 19% of *pilA5* cells lacked T4P, whereas this was observed in only 2% of WT cells. These findings suggest that the absence of one pilus type is not compensated by an increased production of the other.

To investigate the architecture of the T4P machinery in the *pilA5* mutant, we performed sub-tomogram averaging of non-piliated and piliated complexes. Multiple symmetries have been proposed for secretins^9,10,12^, including within the same species^11^. A 7 Å single-particle cryoEM map of detergent-solubilised *Thermus* PilQ reports a 13-fold arrangement^13^. We therefore applied C13 symmetry to the region corresponding to PilQ for subsequent sub-tomogram averaging.

The T4P assembly machinery must overcome symmetry differences between the 6-fold cytoplasmic ATPases^31^ and the higher-order oligomeric state of the PilQ secretin. Such differences are common in large dynamic molecular assemblies such as the bacterial flagellum^32^ and type II secretion systems^33^. The structure of the cytoplasmic membrane assembly platform is unknown. To investigate its potential symmetry, we predicted the structure of PilM, PilN and PilO at varying stoichiometries using AlphaFold3^34^ (Supplementary Figs 4, 5). The predictions support a 6-fold arrangement and place the N-terminus of PilN in association with the cytoplasmic PilM (Supplementary Fig. 4a inset), a result consistent with previous experimental observations^25,27^ and the symmetry of the ATPases^31^. We therefore applied C6 symmetry to the region containing the cytoplasmic-proximal C1 density for subsequent sub-tomogram averaging.

**Fig. 4:**
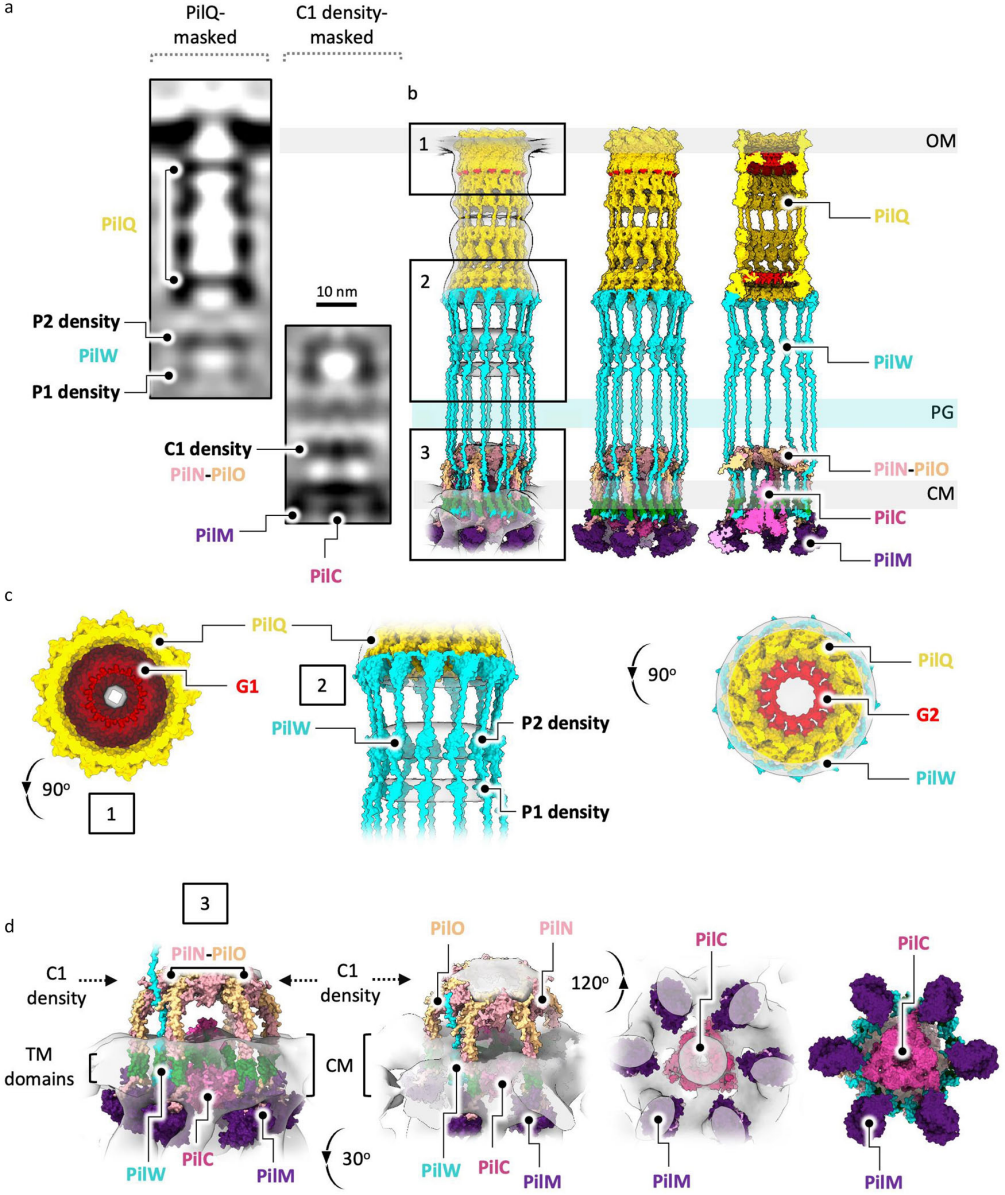
Hypothetical model of the T4P machinery in the non-piliated state. **a** Slices through PilQ-masked and C1 density-masked sub-tomogram average maps, aligned at the P1/P2 density, with all protein densities and corresponding protein labels shown. The text colours of the protein names match the colours of the corresponding structures in (**b**). **b** Hypothetical models docked into the non-piliated state sub-tomogram average maps, from left to right: complete model in the maps, model alone without the maps, and a central slice of the model revealing the gates in PilQ (red). Numbered boxes indicate views enlarged in (**c**, **d**), focusing on: (1) the upper gate (G1) of PilQ, (2) the P1/P2 density (PilW), and (3) the cytoplasmic membrane assembly platform PilM, PilN, PilO and PilC. Different rotated views are shown. OM, outer membrane; PG, peptidoglycan; CM, cytoplasmic membrane; TM, transmembrane domain (green).

Further details on the predictions and the rationale for fitting proteins into the sub-tomogram average map are provided in Supplementary Information Note 2 “Model building of the T4P machinery non-piliated state.” In summary, we fitted the existing closed state PilQ homology model^13^ into the periplasmic density extending from the outer membrane (Supplementary Fig. 6a, b). For the cytoplasmic membrane assembly components, the predicted PilMNO complex suggests that the β-sheet domains of PilN and PilO associate to form a curved structure (Supplementary Fig. 7a). The completed heterohexameric PilNO ring comprises a 48-strand β-barrel built from 4 strands contributed by each PilN and PilO subunit (Supplementary Fig. 7b). The 12 α-helices of PilN and PilO are positioned such that their transmembrane domains are embedded in the cytoplasmic membrane, and their β-sheet domains align appropriately to reach the C1 density (Supplementary Fig. 7c, d), consistent with the protein arrangement and ring architecture observed in other species^12,24^. A trimer of PilC was placed into the cytoplasmic membrane, where the spacing between the membrane-bound domains of PilN and PilO is sufficient to accommodate this arrangement (Supplementary Fig. 8). We propose that PilW bridges PilQ and PilMNO (Supplementary Fig. 9), providing a structural link that may stabilise the assembly and coordinate interactions between outer and cytoplasmic membrane components. The final model shows the closed state PilQ secretin embedded in the outer membrane at its C-terminal domain, linked at the N-terminus to the flexible PilW (P1 and P2), which is in turn linked to the cytoplasmic membrane assembly platform comprised of PilM, PilN, PilO (C1) and PilC (Fig. 4, Supplementary Fig. 15a, b).

### T4P assembly

Pilins possess a hydrophobic N-terminal domain that anchors them in the cytoplasmic membrane prior to incorporation into the growing filament. Interestingly, recent work in *P. aeruginosa* suggests that the dimensions of a pilin subunit and the spacing of the cytoplasmic membrane assembly components would allow diffusion of pilins through the complex to enable incorporation into the growing filament^9^. Given the ~30 Å width of the PilA4 pilin subunit and the ~60 Å spacing between adjacent PilNO complexes in our hypothetical model (Supplementary Fig. 11), it is plausible that a similar mechanism is present in *T. thermophilus*.

Once pilins have reached the assembly platform, they must be incorporated into the T4P. To investigate this, we also built a hypothetical model of the piliated state and conducted molecular dynamics simulations to assess interaction of high-resolution T4P structures with the PilQ homology model. Further details are provided in Supplementary Note 3 “Model building of the T4P machinery piliated state”. In brief, a model of the open state of PilQ was fitted into the corresponding density in the sub-tomogram average (Supplementary Fig. 6c). The position of a PilA4 pilin subunit was predicted at the centre of the PilC trimer (Supplementary Fig. 12a, b), which in turn interacts with a hexamer of the PilF ATPase (Supplementary Fig. 12c). This arrangement resembles the previously proposed hypothetical model of the T4P machinery in *S. sanguinis*^5^ and is consistent with the experimentally verified trimeric architecture of the PulF platform protein in the homologous T2SS, which is predicted to interact with a hexamer of the PulE ATPase^35^.

**Fig. 5:**
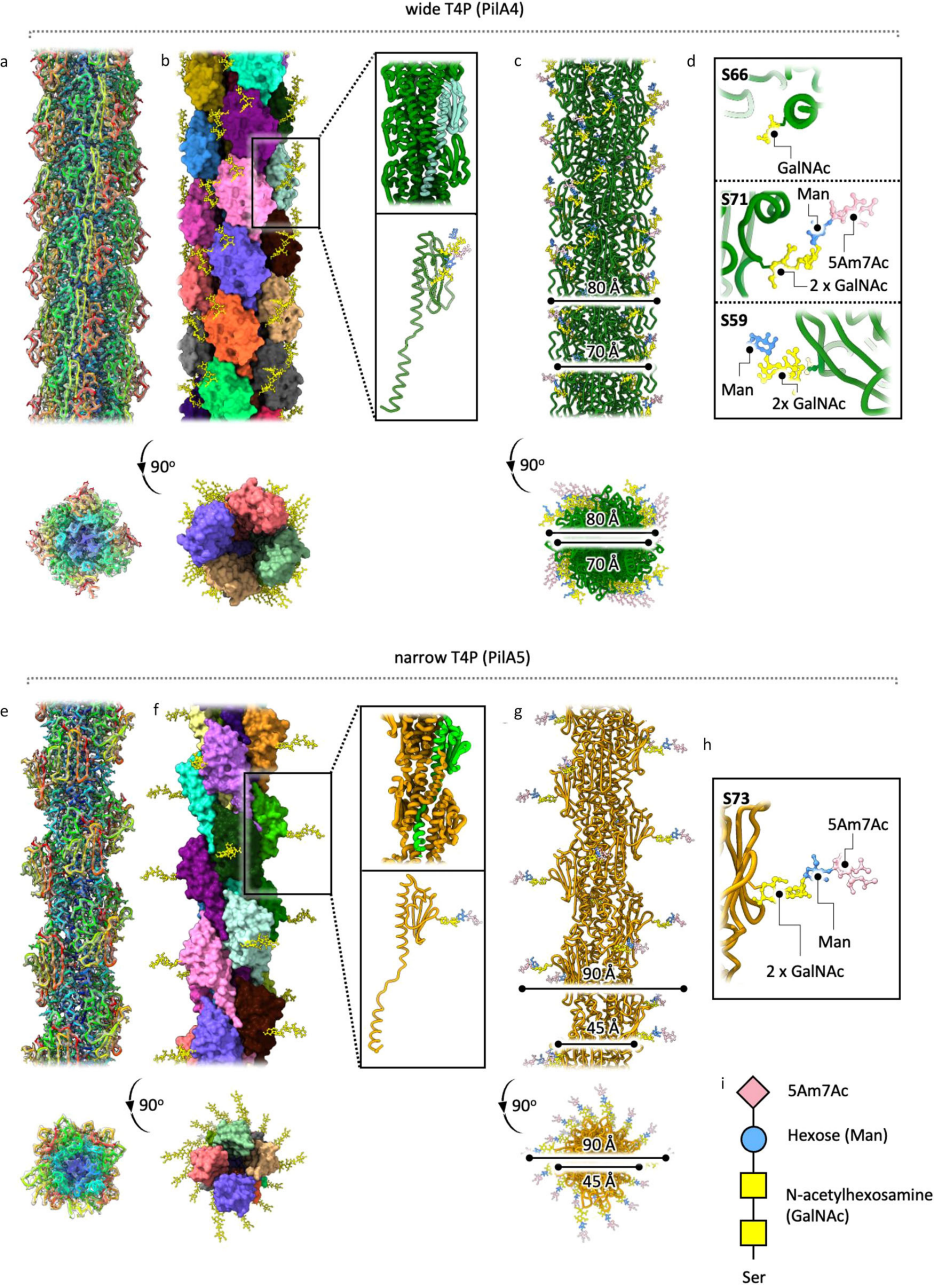
Structures of glycosylated wide and narrow T4P. CryoEM maps and models of T4P comprised of **a**, **b** wide (PilA4) T4P and **e**, **f** narrow (PilA5) T4P, shown in side and 90^o^ rotated views. **a**, **e** Maps (grey) with corresponding rainbow representation models and **b**, **f** models in surface view coloured by subunit, with glycans highlighted in yellow and a single subunit boxed and enlarged. **c**, **g** T4P models with glycans coloured by type, according to the key in (i); **d**, **h** Enlarged views of individual glycan attachment sites (wide: Ser59, Ser66, Ser71; narrow: Ser73).

We had previously determined structures of both wide and narrow T4P by single-particle cryoEM, at resolutions of 3.22 Å and 3.49 Å, respectively^8^. Building on our earlier data and using significantly improved developments in image processing software, we determined new maps at higher resolutions: 2.4 Å (wide PilA4 pilus) and 2.6 Å (narrow PilA5 pilus) (Fig. 5, Table 1, Supplementary Figs. 13a, 14a). Atomic models were refined against the new maps with helical parameters of 92.4° twist, 9.2 Å rise (wide) and 84.0° twist, 11.3 Å rise (narrow), marginally different to the previously determined values (details in Methods).

**Table 1:** CryoEM data collection and model refinement statistics for wide and narrow pili

	Wide PilA4 pilus (EMD-18588) (PDB 8QQD)	Narrow PilA5 pilus (EMD-18593) (PDB 8QQJ)
Data collection and processing		
Magnification	130k	130k
Voltage (kV)	300	300
Electron exposure (e–/Å^2^)	47.56	47.56
Defocus range (μm)	−4.0 to −1.5	−4.0 to −1.5
Pixel size (Å)	1.048	1.048
Helical parameters	Twist: 92.4° Rise: 9.2 Å	Twist: 84.0° Rise: 11.3 Å
Initial segments (no.)	2,763,060	2,763,060
Final segments (no.)	654,248	475,441
Map resolution (Å) FSC threshold	2.40 0.143	2.63 0.143
Refinement		
Initial model used	PDB 6XXD	PDB 6XXE
Model resolution (Å) FSC threshold	2.70 0.5	2.80 0.5
Model refinement resolution (Å)	2.40	2.63
Map sharpening B factor (Å^2^)	0	0
Model composition		
• Non-hydrogen atoms • Protein residues • Glycan monomers	47,025 5625 (125 × 45) 360 (8 × 45)	26,815 3441 (111 × 31) 124 (4 × 31)
B factors (Å^2^)		
• Protein • Glycan	50.5 212.2	69.0 233.6
R.m.s. deviations		
• Bond lengths (Å) • Bond angles (°)	0.008 1.641	0.008 1.582
Validation		
• MolProbity score • Clashscore • Poor rotamers (%)	1.48 4.88 2.0	1.13 1.49 0.0
R.m.s. deviations		
• Favored (%) • Allowed (%) • Disallowed (%)	98.4 1.6 0.0	96.4 3.6 0.0

**Fig. 6:**
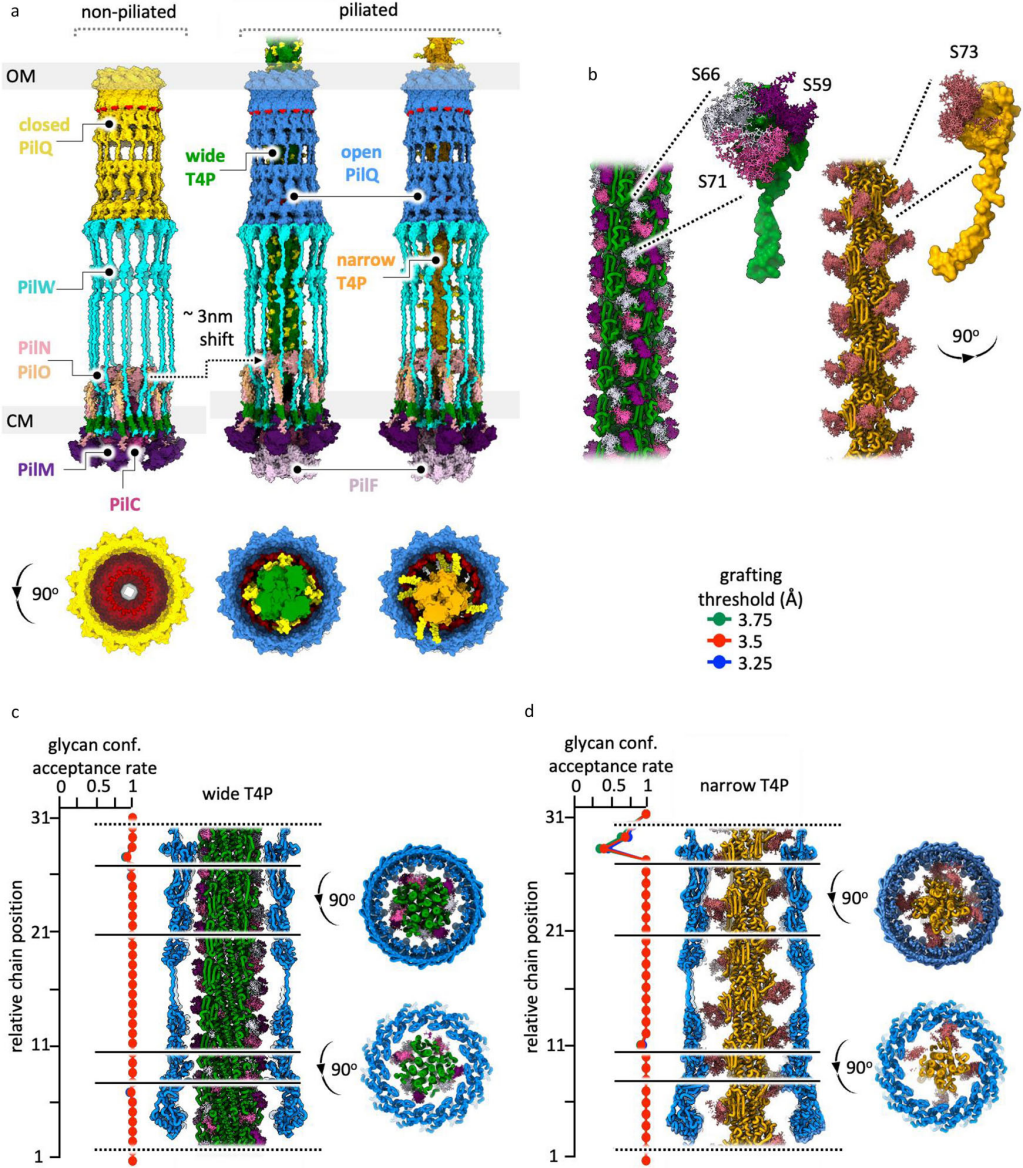
Passage of glycosylated T4P through PilQ. **a** Models of the T4P machinery in non-piliated and piliated states, shown in side and 90° rotated views. The text colours of the protein names match the colours of the corresponding structures, with the closed state of PilQ shown in yellow, the open state in blue, wide T4P (PilA4) in green, and narrow T4P (PilA5) in orange. The PilQ gates are indicated in red. The PilF structure is PDB 6F8L^31^. The dashed arrow indicates an upward shift of the cytoplasmic membrane assembly platform by ~3 nm. **b** T4P models in isolation, with a single subunit extracted and enlarged. Glycans are shown in three shades of purple on the wide T4P and in salmon on the narrow T4P, with the specific glycosylated residues (wide: Ser59, Ser66, Ser71; narrow: Ser73) indicated. GlycoSHIELD was used to investigate plausible glycan conformational ensembles on **c** wide and **d** narrow T4P filaments docked into PilQ. Glycans are coloured as in (**b**). Plots on the left show the rates of glycan conformer rejection due to steric hindrance by PilQ along the length of the docked filaments. Different grafting threshold distances (above panel **d**) were used as proxies to mimic lower (3.25 Å) or higher (3.75 Å) filament stiffness.

In our earlier work, we used mass spectrometry to identify the most abundant glycans^8^, and the improved high-resolution maps now allowed the sugars to be modelled onto the T4P structures (Fig. 5, Supplementary Figs. 13b, 14b). Three O-glycosylation sites are located on the wide T4P (S59, S66 and S71), while the narrow T4P has a single site (S73). Although the narrow T4P have smaller diameters at the protein level, their glycans project farther outward, giving the filaments a larger overall diameter (Fig. 5). Both wide and narrow T4P were fitted into the sub-tomogram average map of the piliated state of the machinery and compared to the non-piliated form (Fig. 6a, Supplementary Fig. 15).

Interestingly, we observe a ~3 nm shift of the C1 density towards PilQ and thus an overall reduction in the length of the T4P machinery on filament assembly (Fig. 6a, Supplementary Fig. 16). Such large scale conformational changes were also observed for the WT average^23^ and are similar to those reported for the *V. cholerae* T4P system^24^. The structure of *T. thermophilus* PilF in the presence of ATP analogues revealed a shift of the regulatory domains towards the ATPase domains^31^. Likewise, current models of T4P assembly propose that ATP hydrolysis drives rotation of the hexameric ATPase with an upward shift of the cytoplasmic membrane assembly platform, facilitating the insertion of pilin subunits^5,16^. Our observation of a shortened piliated T4P machinery may reflect a state in which subunits are actively incorporated into the growing T4P via upward translation from the cytoplasmic membrane.

### Interaction of the T4P filament with the secretin PilQ

Having considered assembly of pilins at the cytoplasmic membrane, we next turned our efforts to understanding how the mature filaments engage with PilQ and are extruded to the external environment. Docking our experimental high-resolution structures of both wide and narrow glycosylated T4P into the model of open PilQ (Fig. 6a, Supplementary Fig. 15d) shows that the filaments fit within the channel. For both T4P, there are regions where the glycans extend sufficiently far from the protein core that they could contact the interior surface of PilQ. Therefore, we conducted molecular dynamics simulations using GlycoSHIELD^36^ to assess glycan flexibility.

Flexibility analysis of both wide and narrow T4P docked into open PilQ showed that all possible glycan conformations on the wide T4P can be accommodated during filament extrusion (Fig. 6c). However, in the narrow filament, a single point in the PilQ C-terminal domain forms an aperture too narrow that only ~50% of glycan conformations can be accommodated (Fig. 6d). During T4P assembly, the single glycan chain on the narrow pilus may need to be constrained closer to the filament core, only fully extending after extrusion to the cell exterior. Evolution thus could have favoured filament flexibility over increasing pore size, which would require more protein subunits and higher energy investment for protein synthesis, folding and membrane insertion. This system could therefore offer an efficient solution, maintaining assembly of two different T4P types with the lowest possible energetic cost.

To provide an indication of glycan flexibility post extrusion, simulations of isolated filaments reveal that the glycans can adopt myriad positions surrounding the central structure (Fig. 6b). As the wide filament contains three times more glycosylation sites than the narrow, the sugars appear as a cloud around the soluble domain of the pilin. The position and extension of the sugar on the single site for the narrow pilus results in glycan positioning which is much more focussed on the exterior surface of the filament. It is well established that T4P mediate diverse biological functions, and our earlier work suggested that the two filaments may serve unique roles^8^. The observed differences in glycan composition likely reflect functional specialisation, although further investigation is required.

## Conclusion

In this work, we employ in situ cryoET, mutagenesis, and AlphaFold predictions to propose hypothetical models of the T4P assembly machinery in non-piliated and piliated states, capturing the overall organisation of the complex. We propose that PilW acts as a dynamic connector that facilitates T4P extrusion by guiding T4P from the cytoplasmic membrane assembly platform towards the PilQ secretin. Furthermore, high-resolution T4P structures reveal extensive glycosylation patterns on two distinct T4P types, providing insight into their structural flexibility. Together, these findings provide a framework for future studies on the structure and function of the T4P assembly machinery.

## Materials and methods

### Strains and culture conditions

*T. thermophilus* HB27 was grown in TM^+^ medium (8 g/l tryptone, 4 g/l yeast extract, 3 g/l NaCl, 0.6 mM MgCl_2_ 0.17 CaCl_2_)^37^. Antibiotics were added when appropriate (kanamycin, 40 μg/ml in solid media (containing 2% agar [w/vol]) and 20 μg/ml kanamycin in liquid media)). The *pilA5::kat*, *pilM::kat*, *pilN::kat*, *pilO::kat* and *pilWΔ163-216* mutants were generated in previous work as described^14,22,38^. *kat* refers to a thermostable kanamycin nucleotidyl transferase, developed in *Thermus* as a selectable marker conferring kanamycin resistance^14^. For negative stain electron microscopy, overnight liquid cultures were diluted to an OD of 0.1 and incubated at 68 °C until an OD of 0.6–0.8 was reached. For cryoET, cells from a 24-h pre-culture were transferred onto TM^+^ plates (containing 2% [wt/vol] agar) and incubated for 48 h at 68 °C.

### Negative stain electron microscopy

Three microliters of exponential phase cell culture were pipetted onto glow-discharged carbon-coated Cu 400 mesh support grids (Sigma-Aldrich) for 2 min. Grids were blotted with Whatman No 41 filter paper and stained with 5% ammonium molybdate for 60 s. Images were recorded with a 120 kV Tecnai Spirit microscope (ThermoFisher Scientific, Waltham, USA) and a OneView CMOS camera (Gatan, Pleasanton, USA). Images were analysed for pilus quality, size, sample density and homogeneity using EMAN2^39^. For the quantitative analysis of number and type of pili per cell, filaments from each cell pole were counted and helices of equal length were selected using e2helixboxer (EMAN2) and subsequently classified in 2D using RELION^40^. The percentage of wide, narrow and unassigned pili was calculated based on the number of particles in each class.

### Sample preparation for CryoET

To determine structures of the T4P machinery, cubes of agar with different *T. thermophilus* mutant cells growing were cut out and placed into buffer containing 20 mM Tris pH 7.4, 100 mM EDTA and gently agitated for 1 h at room temperature. Samples were mixed 1:1 with 10 nm protein A-gold (Aurion, Wageningen, The Netherlands) as fiducial markers. Glow-discharged R2/2 Cu 300 mesh holey carbon-coated support grids (Quantifoil, Jena, Germany) were dipped into the solution without a pipetting step to preserve pili. Grids were blotted for ~4 s in a humidified atmosphere and plunge-frozen in liquid ethane using a Vitrobot Mark-IV (ThermoFisher Scientific). Grids were maintained under liquid nitrogen and transferred into the electron microscope at liquid nitrogen temperature.

### CryoET

Energy-filtered tilt series of T4P mutant strains were typically collected from +60° to −60° at tilt steps of 2° and 2.5–8 μm underfocus, using either a 200 kV Talos Arctica (at the GW4 Regional Facility for CryoEM) or a 300 kV Titan Krios microscope (at the electron Bio-Imaging Centre (eBIC), Diamond Light Source). Data for the WT complex were collected on a Titan Krios at the Max Planck Institute of Biophysics, Frankfurt am Main, Germany, in our previous work^23^. All microscopes were equipped with field-emission guns and Quantum energy filters (Gatan, Pleasanton, USA) operated at a slit width of 20 eV, and either K2 or K3 Summit direct electron detector cameras (Gatan, Pleasanton, USA). Dose fractionated data (3–5 frames per projection image) collected in this work were obtained using TOMO software (ThermoFisher Scientific) at a pixel size of typically 2.8 Å in the Talos or a pixel size of 2.3 Å or 2.7 Å in the Krios. The total dose per tomogram was ~120e^−^/Å^2^. Tilt series were aligned using gold fiducial markers and tomograms reconstructed by weighted back-projection using batch processing in IMOD software^41^ with 2-fold or 3-fold binning. Contrast was enhanced by non-linear anisotropic diffusion (NAD) filtering in IMOD^42^ for visualisation and segmentation of volumes.

### Subtomogram averaging

To facilitate comparisons between mutants of the T4P machinery relative to our previously published work on the WT, we followed a similar workflow^23^. Coordinates corresponding to the outer membrane and cytoplasmic membrane domains of the complex were marked manually in IMOD. Subvolumes were then extracted from NAD filtered data and an initial alignment and averaging performed in SPIDER^43^. This average was used as a reference for alignment and refinement using PEET^44^. A single-particle cryoEM structure of PilQ purified from *Thermus* suggests 13-fold symmetry^13^. Thus, we applied 13-fold symmetry to the PilQ secretin by 27.7° (360°/13 subunits) rotation of each subvolume prior to the alignment search using a mask drawn around PilQ. Resolution estimates are an output from PEET and reported at the 0.5 criterion (Supplementary Table 1).

To generate sub-tomogram average maps used for model building of the non-piliated and piliated forms of the machinery, we employed a more recent version of PEET that allows symmetry to be applied without manual symmetry expansion. Subvolumes (33 for the non-piliated and 24 for the piliated complex) from 32 tomograms were extracted as before and reconstructed with 13-fold symmetry. Iterative refinements masking around the PilQ region were performed. The motor ATPases have 6-fold symmetry^31^, thus these steps were then repeated around the cytoplasmic membrane coordinates with 6-fold symmetry and a mask applied around the C1 density. The particles were then split into half-sets and refined separately to allow resolution estimates to be obtained in RELION. The half-maps were normalised and processed with the RELION-4 command^45^ relion_tomo_taper, then post-processed using the relion_postprocess command through generation of half-maps. Resolution estimates are reported at the 0.143 criterion (Supplementary Fig. 17a). The resulting non-piliated and wide-piliated sub-tomogram averages of the machinery in the *pilA5* mutant resemble that of the WT determined in our earlier work^23^, confirming the consistency between the data processing approaches (Supplementary Fig. 18). UCSF Chimera^46^ was used to draw all surface views and remove low-contrast background noise using the ‘hide dust’ tool.

### Helical reconstruction

The patch correction package from cryoSPARC was used to align the frames of 2455 movies containing both wide and narrow T4P. Defocus variation was estimated using the patch CTF estimation program. The native manual picker within cryoSPARC was used to manually pick either wide or narrow filaments, which were used as templates for the filament tracer programme. Iterative rounds of 2D classification were performed to classify the two filament types and remove poorly aligned particles.

A total of 2,763,060 particles were subjected to 3D refinement using the helical parameters previously determined (9.3 Å rise and 92.5° twist for the wide T4P, and 11.3 Å rise and 84.3° twist for the narrow T4P)^8^. Reference-based motion correction, global and local CTF refinement jobs were performed prior to final helical refine, ultimately leading to global resolutions of 2.4 Å and 2.6 Å for the wide and narrow T4P respectively (Table 1, Supplementary Fig. 17b). For the final reconstruction, 475,441 particles were used for the narrow T4P, while 654,248 particles were used for the wide T4P. Resolutions were estimated using Fourier shell correlation between two sets of independently refined half sets, using the Gold Standard correlation value of 0.143. To validate the helical parameters, completely unbiased reconstructions were performed without any symmetry applied, and by relaxing the symmetry at the final reconstruction step. All maps were overlaid and were in agreement. The improvement in resolution resulted in very slightly altered helical parameters: 9.2 Å rise and 92.4° twist (wide T4P) and 11.3 Å rise and 84.0° twist (narrow T4P). We were also able to identify a leucine at position 67 in PilA5, which was previously assigned as a proline. Both residues are common in this position across *Thermus* species (Supplementary Fig. 19), and alteration of the residue does not affect the overall structure. Local resolutions were calculated within cryoSPARC, and the maps visualised in ChimeraX.

### Glycan modelling

The new T4P atomic models were used as a framework onto which to model the glycans. In our previous work^8^, we used mass spectrometry to reveal that the two most abundant glycans are tetrasaccharides W-H-N-N-(Ser) and X-H-N-N-(Ser), where N is N-acetylhexosamine, H is hexose, W, Pseudaminic acid derivative 5Am7Ac (7-Acetamido-5-acetimidoyl-3,5,7,9-tetradeoxy-L-glycero-L-manno-nonulosonic acid) and X is an unknown and previously unreported monosaccharide with a molecular mass of 346 Da^8^. We also conducted lectin analysis of isolated pili to show that the N-acetylhexoasamine is a-N-acetylgalactosamine (α-GalNAc) and the hexose is either α -mannose (Man) or α-glucose (Glc). No information is available regarding the connectivity within the tetrasaccharide.

The tetrasaccharide 5Am7Ac-α(1-4)Man-α(1-3)GalNAc-α(1,3)GalNAc-α-Ser was built into the density attached to S71 of the wide T4P using Coot^47^. A refinement dictionary entry for 5Am7Ac was created in JLigand^48^. The density map suggested glycolytic α(1-3) links between the two GalNAc moieties and GalNAc-hexose. The available density also favoured positive chirality at C2 of hexose, thus choosing Man over Glc. 5Am7Ac-α(1-4)Man-link was also the best fit, although α(1-3) and α(1-6) cannot be ruled out. The density attributed to glycans linked to S59 and S66 on the wide pilus is less well defined, therefore only a trisaccharide of α(1-4)Man−α-GalNAc-α(1,3)GalNAc was built attached to S59, and a single GalNAc was built linked to S66. In narrow pili the full-length tetrasaccharide attached to S73 was fitted. The structures were refined with Phenix^49^.

### Molecular dynamics simulations

Conformation arrays of full-length glycan chains were grafted onto the T4P using GlycoSHIELD^36^. In brief, glycans attached to ASA tripeptides were modelled in CHARMM-GUI^50^ and solvated using a TIP3P water model^51^ in the presence of 150 mM NaCl and configured for simulations with CHARMM36m force fields^52,53^. Pseudaminic acid (5Am7Ac) is not yet available in CHARMM-GUI and was approximated and replaced by Neuramimic acid (Neu5Ac) in the modelled tetrasaccharides. Simulations were performed with GROMACS 2020.2 and 2020.4^54^ in mixed GPU/CPU environments. Potential energy was first minimized (steepest descent algorithm, 5000 steps) and systems were equilibrated in NVT ensemble (constant number of particles, N, volume, V and temperature, T) with 1 fs time-steps using Nose-Hoover thermostat^55^. Atom positions and dihedral angles were restrained during the equilibration, with initial force constants of 400, 40 and 4 kJ/mol/nm^2^ for restraints on backbone positions, side chain positions and dihedral angles, respectively. The force constants were gradually reduced to 0. Systems were additionally equilibrated in NPT ensemble (constant number of particles, pressure, P and temperature, T with Parrinello-Rahman pressure coupling with the time constant of 5 ps and compressibility of 4.5 10^−5 ^bar^−1^ over the course of 10 ns with a time step of 2 fs. Hydrogen bonds were restrained using LINCS algorithm^56^. During the production runs, a velocity-rescale thermostat^57^ was used and the temperature was kept at 351 K. Production runs were performed for an aggregated duration of 3 μs and snapshots of atom positions stored at 100 ps intervals. Glycan conformers were grafted using GlycoSHIELD with a threshold distance of 3.25 to 3.75 Å between protein α-carbons and glycan ring-oxygens. Glycan conformers were shuffled and subsampled for analysis and to represent plausible conformations in the displayed renders. Graphics were generated with ChimeraX^58^.

### Model building

A detailed description for building the hypothetical model of the T4P machinery is given in the Supporting Information.

### Statistics and reproducibility

For the data shown in Fig. 3 and Supplementary Fig. 2, images are representative from ~200 and the findings are described by quantitative data, *n* = 3 independent biological replicates comprising 128 WT cell poles and 121 wide T4P-expressing cell poles. Plots in Supplementary Fig. 3 are generated from 14 technical replicates. Single-particle data shown in Fig. 5 are based on two independently determined cryoEM maps from a single dataset of 2455 images. Model refinement statistics are shown in Table 1. The number of tomograms collected in the analysis of *pilM::kat*, *pilN::kat*, *pilO::kat* and *pilWΔ163-216* mutants is reported in Supplementary Table 1. The hypothetical model is built from analysis of 32 tomograms collected of the *pilA5::kat* mutant; further details can be found in Materials and Methods.

### Reporting summary

Further information on research design is available in the Nature Portfolio Reporting Summary linked to this article.

## Supplementary information

Supplementary Information

Description of Additional Supplementary Files

Supplementary Data 1

Reporting Summary

## Data Availability

The subtomogram averages used to build the hypothetical models have been deposited in the Electron Microscopy Data Bank (EMDB; https://www.ebi.ac.uk/emdb/) under accession codes EMD-56470, EMD-56471, EMD-56468, and EMD-56469 for the non-piliated (closed) C13, non-piliated (closed) C6, piliated (open) C13, and piliated (open) C6 sub-tomogram average maps, respectively. The cryo-EM density maps and atomic coordinates for the wide and narrow T4P have been deposited in the EMDB and Protein Data Bank (PDB; https://www.rcsb.org/) under accession numbers EMD-18588 / 8QQD and EMD-18593 / 8QQJ, respectively. Glycan conformers used for GlycoSHIELD analyses as well as the topology and starting configuration used for glycan simulations have been deposited in Zenodo (10.5281/zenodo) under the accession number 18488813. Source data underlying the graphs in Fig. 3b, c, and Supplementary Fig. 3c, d and e are provided in Supplementary Data 1.
