# Supplementary material for: Structural insights into the Thermus thermophilus type IV pilus machinery assembling two distinct pili
Source: Commun Biol. 2026 Mar 31;9:474. doi: 10.1038/s42003-026-09762-0 (PMC13039148; doi:10.1038/s42003-026-09762-0)
Supplement: Supplementary file 1 — Supplementary Information [file 42003_2026_9762_MOESM1_ESM.pdf]

Supplementary Information

Structural Insights into the *Thermus thermophilus* Type IV Pilus Machinery Assembling Two Distinct Pili

Alexander Neuhaus, Mathew McLaren, Michail N. Isupov, Matthew Gaines, Emma Buzzard, Mateusz Sikora, Cyril Hanus, Bertram Daum, Beate Averhoff & Vicki A. M. Gold.

This file contains:

Supplementary Notes 1-3
Supplementary Figures 1-19
Supplementary Table 1
Supplementary References

Supplementary Note 1

Impact of T4P assembly mutations on complex stability

Caution is required when assessing the impact of mutations on complex stability, given differences in particle numbers, the use of different microscopes for data collection, and the limitations of nanometre-scale models for drawing precise conclusions.

Compared to the WT, PilQ gate densities appear weaker for the PilM and PilN mutants, but comparable for the PilO mutant (Fig. 1b, d, Supplementary Fig. 1), perhaps indicative of different levels of complex stability. In the PilM and PilN mutants, the P1 densities are also more difficult to distinguish from the peptidoglycan layer, suggesting potential additional destabilisation and greater structural flexibility, resulting in poorer definition. These effects may reflect the functional relationship between PilN and PilO, which form homologous heterodimers; however, PilN additionally plays a stabilising role by linking to PilM via its N-terminus[1,2]. Consequently, loss of PilO may be less destabilising than loss of PilN or PilM, resulting in the clearer PilQ, P1, and P2 densities observed in the PilO mutant.

The PilQ gates in the *pilWΔ163–216* mutant are more clearly defined than in the other mutants (Fig. 2c, Supplementary Fig. 1). This is possibly due to the non-piliated *pilWΔ163–216* mutant average comprising approximately twice as many particles as the PilM, PilN and PilO mutants, due to collecting a larger number of tomograms for this strain. We considered this necessary to ensure the confident absence of P1 and P2 densities before proposing these features as a plausible location of PilW.

Supplementary Note 2

Model building of the T4P machinery non-piliated state

All predictions were made using AlphaFold3[3] and models fitted into sub-tomogram average maps using ChimeraX[4].

The non-piliated (closed state) of the PilQ secretin: Our non-piliated (closed state of PilQ) is based on a model obtained from a 7 Å single-particle cryoEM map that suggests 13-fold symmetry[5]. The *in situ* averages revealed a more elongated conformation of PilQ compared to its detergent purified form (Supplementary Fig. 6a). This observation was consistent across both the WT and *pilA5::kat* strains, and with data acquired from different microscopes (Supplementary Table 1). Based on these findings, the following modifications were made to the PilQ model in Coot[6] (Supplementary Fig. 6b): residues 1-359 (domains N0-N3) were translated downwards (away from the OM) into the sub-tomogram average map. Residues 363-421 (domain N4) were tilted outwards and downwards slightly around residue 363. Residues 423-505 (domain N5) were tilted outwards slightly around residue 505. Linkers between the domains were adjusted manually and using "regularize zone" in Coot. The model was refined using Refmac[7].

The alignment complex proteins PilM, PilN and PilO: We hypothesised that the alignment complex, comprised of PilM, PilN and PilO, contributes to the C1 density, although its stoichiometry remains unknown. In *Thermus*, PilMNO complexes can be isolated[8] but a structure of the complete assembly is missing. First, we predicted the structure of a complex containing single copies of PilM, PilN and PilO, with the predicted local distance difference test (pLDDT) score showing an overall confident prediction on the per-atom scale, supported by the predicted alignment error (PAE) for the highest-ranking prediction (Supplementary Fig. 4a). The prediction places the N-terminus of PilN in association with PilM (Supplementary Fig. 4a inset), a result consistent with previous experimental observations[1,2].

Values for the interface predicted template modelling (ipTM; a measure of accuracy for predicted relative subunit positions within a complex) and predicted template modelling (pTM; an integrated measure of overall prediction accuracy) for the highest-ranking prediction of PilMNO was 0.58 and 0.55 respectively (Supplementary Fig. 4a). A pTM score above 0.5 is deemed acceptable, whereas the

ipTM score of <0.6 suggests a failed prediction. The prediction outcome is plausibly attributable to the fact that a monomeric unit of PilMNO is not expected to be the native stoichiometry of the complex.

Therefore, we increased the number of units in the PilMNO predictions incrementally up to six copies of each, which at the time of writing, was the largest size it was possible to predict using AlphaFold3 (Supplementary Fig. 4b-f). Of the multimeric structures, the hexamer was predicted at the highest confidence, but nevertheless not deemed an accurate prediction based on the ipTM score of 0.38 and the pTM score of 0.43. The area of lowest confidence on the per-atom scale lies in the long α-helices of PilN and PilO which have been pulled inward in the prediction and show a low pLDDT score (arrowhead in Supplementary Fig. 4f). This observation may be attributable to the fact that the cytoplasmic membrane protein PilC, which potentially localises to this region, was absent from the prediction. We reasoned that as a fully cytoplasmic protein, PilM is unlikely to substantially affect the architecture of the PilNO complex. Therefore, to explore higher stoichiometries, we excluded PilM from the predictions, which allowed us to predict heterodimeric PilNO complexes up to an 8-fold assembly (Supplementary Fig. 5). Once again, the 6-fold arrangement emerged as the most confident prediction, with an ipTM score of 0.5 and a pTM score of 0.53. We therefore applied C6 symmetry for sub-tomogram averaging of the region corresponding to PilM, PilN and PilO, in line with previous experimental observations[1,2] and the symmetry of the ATPases[9].

Interestingly, the predicted complex containing single copies of PilM, PilN and PilO shows the β-sheet domains of PilN and PilO associate to form a curved structure (Supplementary Fig. 7a). As the single copy prediction was the most confident at the all-atom scale (Supplementary Fig. 4a), we used the PilMNO complex as a building block to assemble a PilMNO hexamer, completing the curved β-sheet (Supplementary Fig. 7b). Next, we investigated the positions of the PilN and PilO transmembrane domains using DeepTMHMM[10] (Supplementary Fig. 7c) and positioned these into the density corresponding to the cytoplasmic membrane in the sub-tomogram average (Supplementary Fig. 7d). In this arrangement, the long α-helices of PilN and PilO are positioned at the correct distance to place their β-sheet domains into the C1 density, consistent with the protein arrangement and ring architecture observed in other species[11,12].

An *in situ* model of the *M. xanthus* T4P machinery placed dodecamers of PilM, PilN and PilO into the equivalent C1 position observed in a sub-tomogram average, matching the stoichiometry proposed

for the *M. xanthus* PilQ protein[11]. In *P. aeruginosa*, this ratio is 14 PilQ:7 PilMNO; although the subunit numbers differ, the symmetry aligns[13]. Fitting our hexameric PilMNO model into the C1 density confirmed that any assembly larger than a six-fold arrangement would exceed the dimensions of the map (Supplementary Fig. 7c), while assemblies with fewer than six subunits are unlikely given the C6 symmetry of the associated ATPase[9].

The alignment complex protein PilC: PilC or its homologues have been proposed to form both dimers[11,14–17] or trimers[13,18,19]. However, no high-resolution structural evidence is currently available to confirm either oligomeric state. Thus, we used AlphaFold to predict the structure of both dimeric and trimeric forms (Supplementary Fig. 8a, b). The N-terminal 38 residues are disordered and were excluded from the prediction, as their inclusion substantially reduced the confidence scores. The trimeric form was predicted at a reasonable degree of confidence (ipTM of 0.62 and pTM of 0.66) whereas the dimeric form was not reliably predicted (ipTM of 0.43 and pTM of 0.56). Based on this reasoning, we propose that the trimer represents the most likely stoichiometry for PilC in *Thermus*, in conjunction with a hexamer of PilM, PilN and PilO. However, at the time of writing, a multimeric PilMNOC complex contains too many residues for prediction using AlphaFold.

Next, we investigated the positions of the PilC transmembrane domains using DeepTMHMM[10]. We used these findings as a guide to place the predicted transmembrane domains into the density in the subtomogram average corresponding to the cytoplasmic membrane, where the cytoplasmic domains of PilC fit inside the PilM ring (Supplementary Fig. 8c). We placed the trimeric PilC complex into the sub-tomogram average map of the non-piliated (closed) T4P machinery (Supplementary Fig. 8d).

PilW: To investigate if PilW comprises the P1 and P2 densities, we again used AlphaFold to predict the structure as there are no experimental data available. PilW has a hydrophobic N-terminus which may form a membrane anchor[20] and has been found equally distributed between inner and outer membrane fractions, which may suggest a bridging role for the protein between PilQ and the cytoplasmic membrane alignment complex[20,21]. PilW does not display sequence similarity to other known proteins[20]. However, *pilP* is found in the same chromosomal location as *pilW* relative to other *pil* genes[22], and has been shown to fulfil a similar role, its C-terminal domain binding PilQ to the cytoplasmic membrane components via its N-terminal domain[23].

AlphaFold predicted an overall low confident structure for PilW, with the protein exhibiting significant disorder (Supplementary Fig. 9a). We therefore predicted the structure of PilW in complex with proteins it is likely to interact with. We reasoned that to bridge PilQ with the PilMNO alignment complex, different regions of PilW would interact with different partner proteins. First, we predicted the structure of the PilQ N-terminal domain (N0, residues 1-116), which projects furthest into the periplasm, in complex with either the N- or C- terminal domains of PilW (Supplementary Fig. 9b, c). The prediction of PilQ with the N-terminal domain of PilW (residues 1-50) was predicted at low confidence (Supplementary Fig. 9b), with ipTM and pTM scores of 0.13 and 0.56 respectively. In contrast, the C-terminal domain of PilW (residues 216-292) showed a high confidence interaction with PilQ (Supplementary Fig. 9c), with ipTM and pTM scores of 0.71 and 0.62 respectively, suggesting that there may be an interaction between these domains. This supports the hypothesis that the N-terminal domain of PilW could locate to the cytoplasmic membrane[20] and form a membrane anchor with PilMNO. To test this, we predicted the structure of the PilW N-terminal domain in complex with one copy each of PilM, PilN and PilO (Supplementary Fig. 9d). Interestingly, AlphaFold predicted that the PilW N-terminus (identified as the membrane anchor by DeepTMHMM[10]) could form a helical bundle with the α-helices of PilN and PilO, which are embedded in the cytoplasmic membrane in our model (Supplementary Fig. 9e). The findings support a bridging role for PilW between PilQ and PilMNO, consistent with experimental observations[20,21].

The density corresponding to P1 and P2 cannot be explained by a fully extended PilW model, as the unstructured amino acid chain would be too long and insufficient to account for the observed mass. The density in these regions corresponds to PilW residues ~111-195, which display a pattern of regularly spaced opposing charges (Supplementary Fig. 10a). Such charge distributions may promote intra-chain interactions in the absence of secondary structure[24]. Based on the spatial arrangement of these charges, we modelled this portion of PilW in a more compact conformation devoid of secondary structure (Supplementary Fig. 10b). Nevertheless, we cannot exclude the possibility that additional, as-yet uncharacterised components, may contribute to the P1 and P2 densities.

In line with the hexameric ATPases and fit to the sub-tomogram average maps, we suggest that PilMNO complexes form heterohexamers. However, the stoichiometry of PilW remains unknown. CryoET models obtained from *M. xanthus* propose a 1:1 interaction between PilQ and PilP[11], the functional homologue of PilW[22,23]. In *P. aeruginosa*, the stoichiometry is proposed to be 2:1, reflecting a defined interaction pattern analogous to the 1:1 association and incompatible with an odd-

numbered oligomer[13]. Given that current data suggest that *Thermus* PilQ forms an odd-numbered oligomer[5], based on current data it is difficult to reconcile these observations with a stoichiometry other than 1:1. This interpretation is further supported by reports of 1:1 stoichiometry between PilQ and different functional homologues of PilW using complementary experimental approaches[25,26].

Accordingly, we modelled 13 copies of PilW into the C13 sub-tomogram average, guided by the predicted interaction between the C-terminal domain of PilW and PilQ (Supplementary Fig. 9c). The unstructured regions of PilW are sufficiently long to bridge PilQ and the PilMNO complex, providing structural flexibility. This is consistent with our observations (Fig. 2d) and may be facilitated by the high proline content of the protein, which is known to disrupt regular secondary structure. This flexibility likely explains both the absence of visible linkers in our sub-tomogram average maps and the comparably weaker density observed for PilW relative to PilQ (Fig. 4a, Supplementary Fig. 15).

Supplementary Note 3

Model building of the T4P machinery piliated state

All predictions were made using AlphaFold3[3] and models fitted into maps using ChimeraX[4].

The piliated (open state) of the PilQ secretin: To model the open state of PilQ we employed a method used previously[27]. The open and closed structures of the InvG secretin from *Salmonella typhimurium* (PDB 6Q15 and 6PEE)[28] were superimposed onto the closed state homology model of *Thermus* PilQ and positioned within the sub-tomogram average of the piliated state. Gate 1 was modelled into the equivalent open conformation, following the arrangement observed in 6Q15, by remodelling the loops spanning residues 590-624 and 639-663 (Supplementary Fig. 6c). For Gate2, the N1 domain (residues 119-219) was rotated outwards, and the loop between residues 135 and 145 was tilted upwards. The model was refined using Refmac[7].

PilC-PilA4: It has been suggested that PilC may rotate to add or remove pilin subunits from the membrane[29]. To explore a potential interaction, we predicted the structure of a PilC trimer in complex with a PilA4 pilin subunit (Supplementary Fig. 12a). AlphaFold positioned the monomer at the centre of the PilC trimer, suggesting a possible mechanism by which PilC could add or remove pilin subunits.

PilF: We used the previously determined structure of PilF (PDB 6F8L)[9] as the cytoplasmic ATPase. To align PilF to the T4P machinery, we predicted a complex of a trimer of PilC, a single copy of PilA4, and a hexamer of the C-terminal ATPase domain (residues 477 – 889) of PilF (Supplementary Fig. 12b, c). This 3:6 stoichiometry of PilC:PilF matches that reported for the PulF trimer and PulE ATPase from the related type 2 secretion system[18]. We used the position of the ATPase domain to position the structure of the complete PilF including the regulatory domains GSPII-C and GSPII-B[9] .

T4P comprised of PilA4 or PilA5: Analysis of a complex containing PilW revealed the presence of PilA5, as well as a second complex comprising both PilA5 and PilA4 together with other T4P-associated proteins[21]. A complex of PilN, PilO and PilC was also shown to form heteropolymeric complexes with PilQ that interact with both PilA4 and PilA5 via PilC[21]. Given that PilA4 and PilA5 form distinct filaments[30] and that only a single PilQ secretin is present on the *T. thermophilus* genome, these findings suggest that both T4P pass through the same complex. We used the density

corresponding to the T4P in the sub-tomogram average map, and the position of PilA4 within the PilC trimer, to guide the localisation of both glycosylated T4P filament structures (Fig. 5) into the centre of PilQ (Fig. 6a).

Supplementary Figures

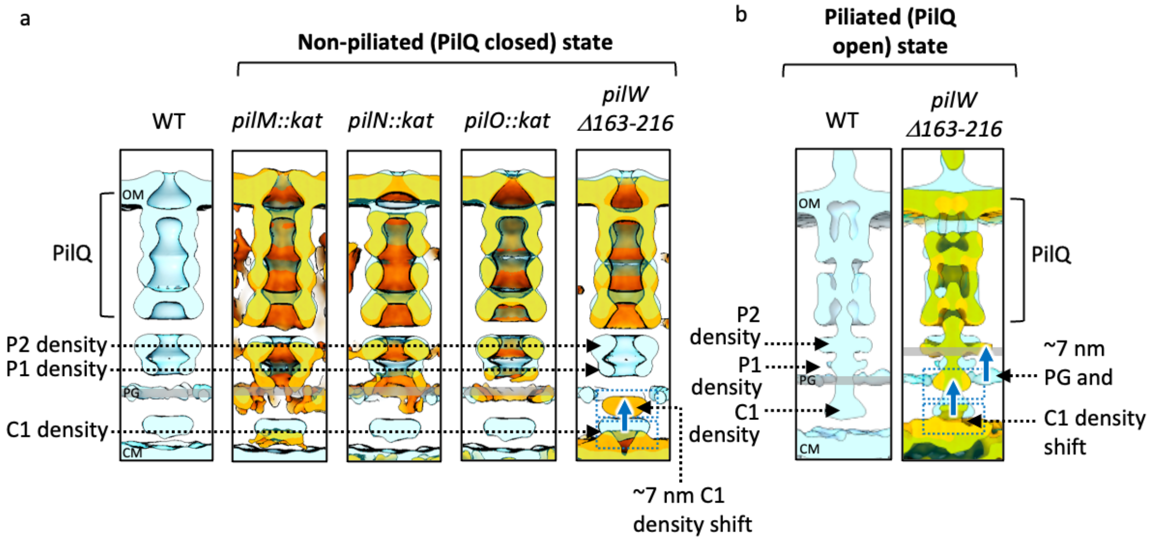

c

	WT	pilM::kat	pilN::kat	pilO::kat	pilW Δ163-216		WT	pilW Δ163-216
**PilQ**	yes	yes less defined	yes less defined	yes	yes		yes	yes
**P2 density**	yes	yes	yes	yes	no		yes	unclear
**P1 density**	yes	yes less defined	yes less defined	yes less defined	no		yes	unclear
**PG**	yes	yes	yes	yes	unclear		yes	shift
**C1 density**	yes	yes less defined	no	no	shift		yes	shift

Supplementary Fig. 1. Comparisons between non-piliated and piliated T4P complexes in mutant strains compared to the WT.

3D volume representations of the WT (blue) overlaid with mutant strains to highlight differences in (a) non-piliated (dark orange) and (b) piliated (pale orange) forms of the machinery. PilQ, P1/P2, and C1 densities are indicated. In the *pilWΔ163-216* mutant, C1 and the peptidoglycan appears shifted ~7nm towards PilQ (blue boxes and arrows). OM, outer membrane; PG, peptidoglycan, CM, cytoplasmic membrane. (c) Observations are summarised in the table below the corresponding data in (a, b).

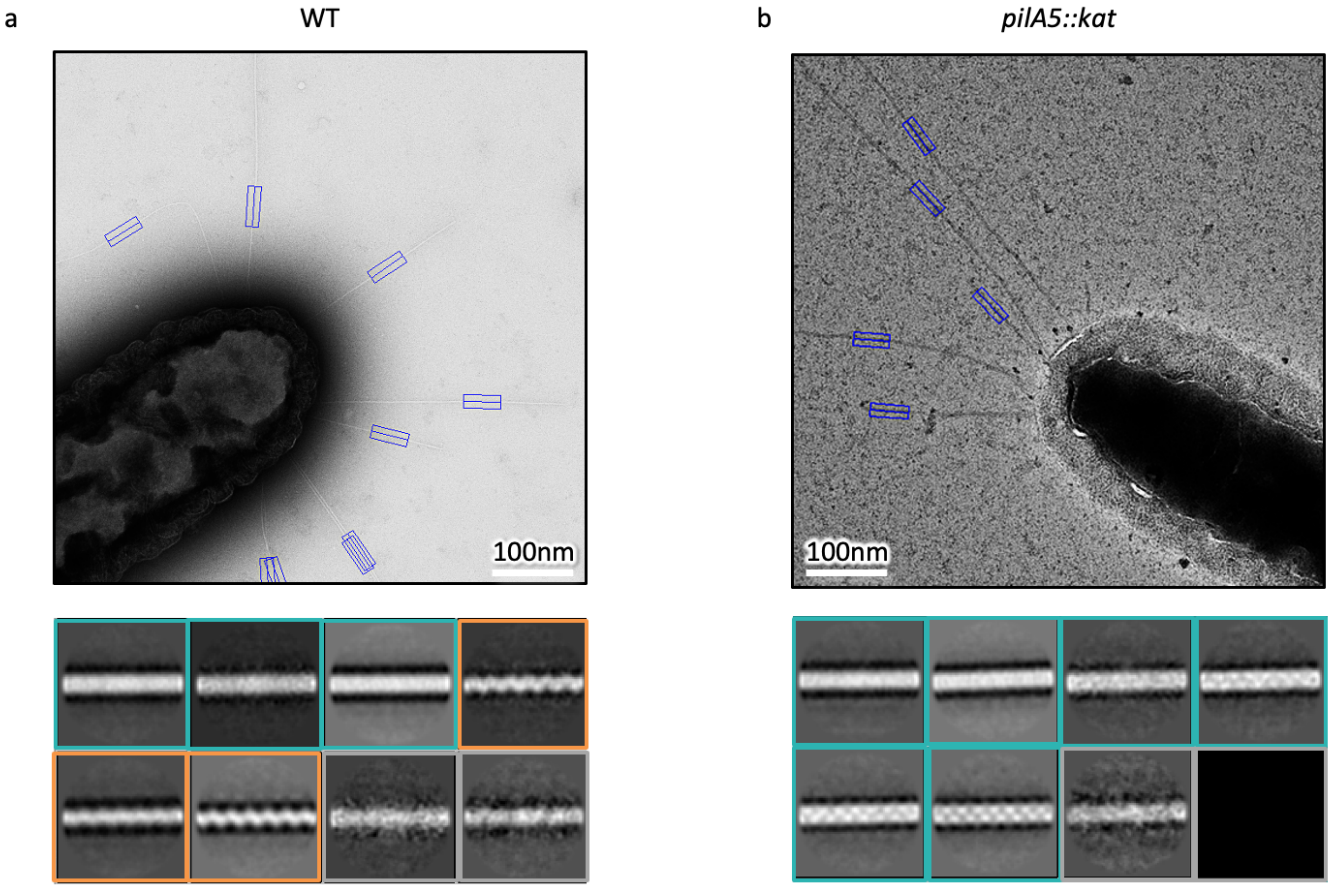

Supplementary Fig. 2. The PilA5 mutant produces only wide T4P

Representative images of negatively stained *T. thermophilus* cells, with regions containing T4P boxed and the corresponding 2D class averages shown below. Teal boxes indicate classes of wide T4P, orange boxes narrow T4P, and grey boxes filaments that could not be clearly classified. The class averages for (a) WT and (b) *pilA5::kat* cells were previously shown in Supplementary Fig. 3 of our earlier work[30]. The article was licensed under a Creative Commons Attribution 4.0 International License, which permits use, sharing, adaptation, distribution and reproduction in any medium or format (https://creativecommons.org/licenses/by/4.0/). n = 3 biological replicates per strain, with a minimum of 35 cell poles imaged per replicate.

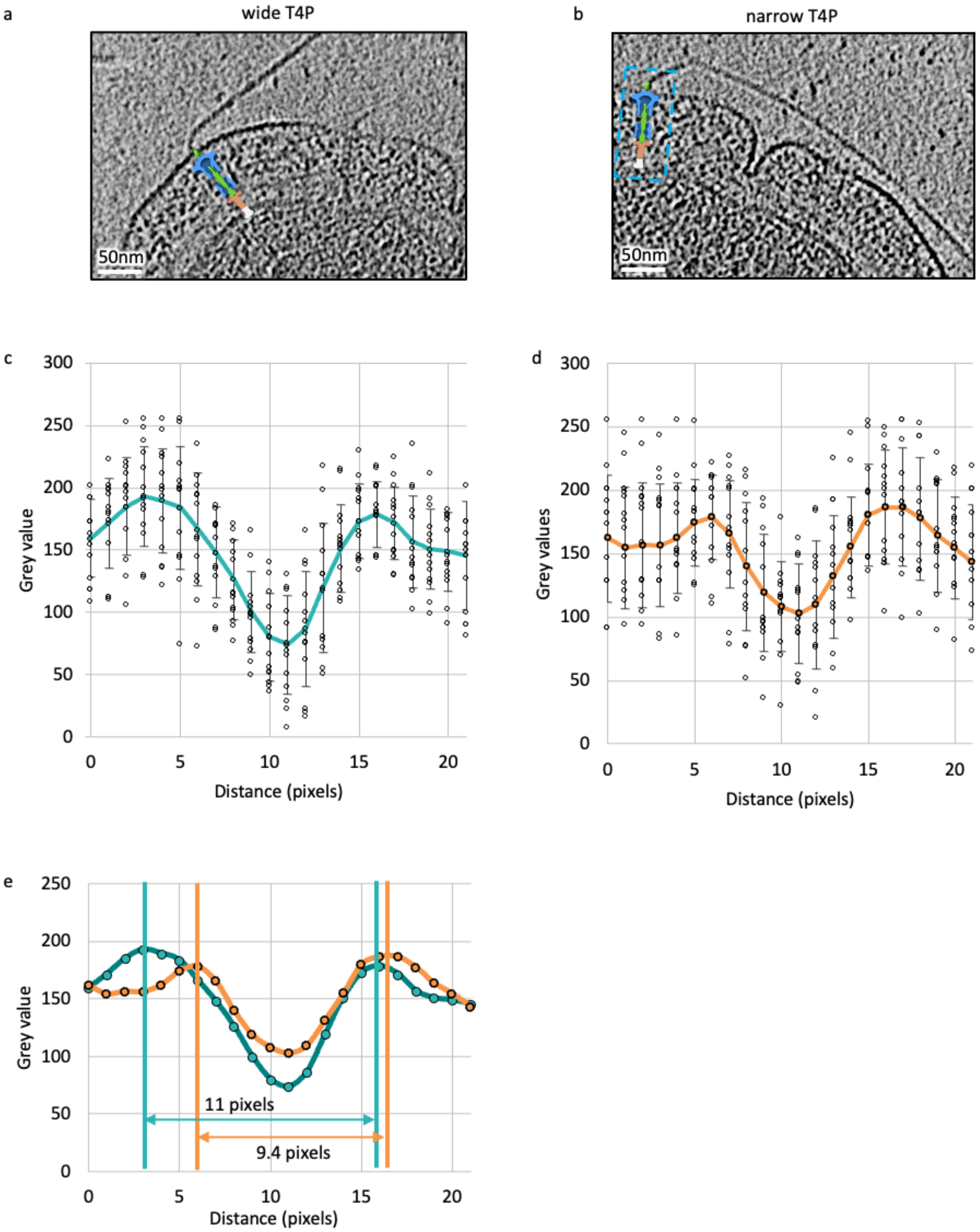

Supplementary Fig. 3. Examples of piliated T4P complexes assembling wide and narrow pili

Slices through cryo-tomograms showing machinery assembling (a) wide and (b) narrow T4P. Corresponding width plot profiles are shown below in (c, d) with an overlay in (e) for comparison. The wide T4P is shown with a teal trace and narrow with an orange trace. n = 14 technical replicates.

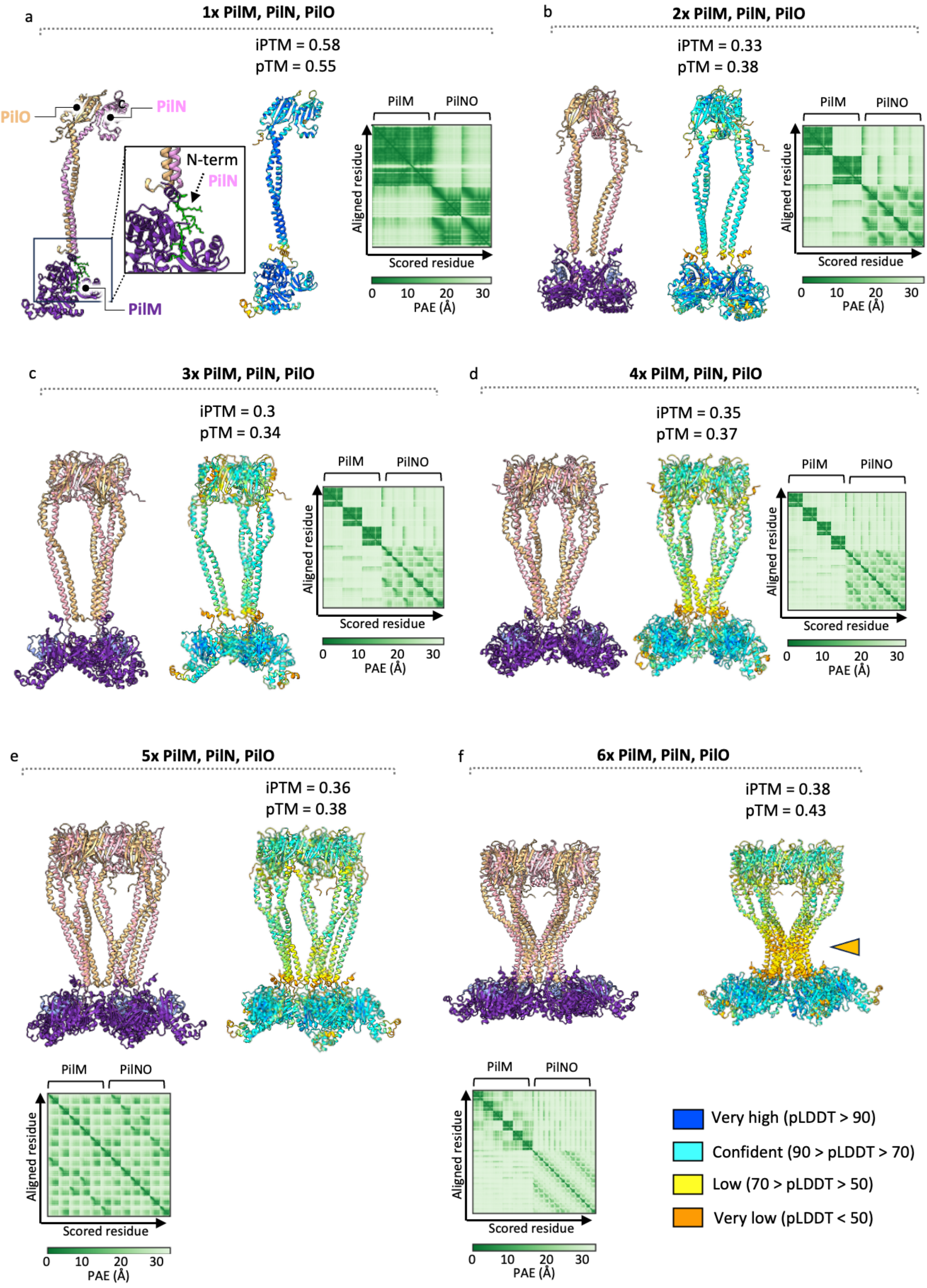

Supplementary Fig. 4. AlphaFold predictions of PilMNO

(a) AlphaFold prediction for a complex of PilM, PilN and PilO with each protein coloured differently and by per residue pLDDT score according to the key (bottom right), with corresponding ipTM and pTM values and predicted alignment error (PAE). The boxed region shows an enlarged view of the N-terminus of PilN, with side-chains (green) interacting with PilM (purple).

(b – f) Predictions with increasing numbers of PilM, PilN and PilO subunits. Per-residue pLDDT scores are shown according to the key (bottom right), with corresponding ipTM and pTM values and predicted alignment error (PAE).

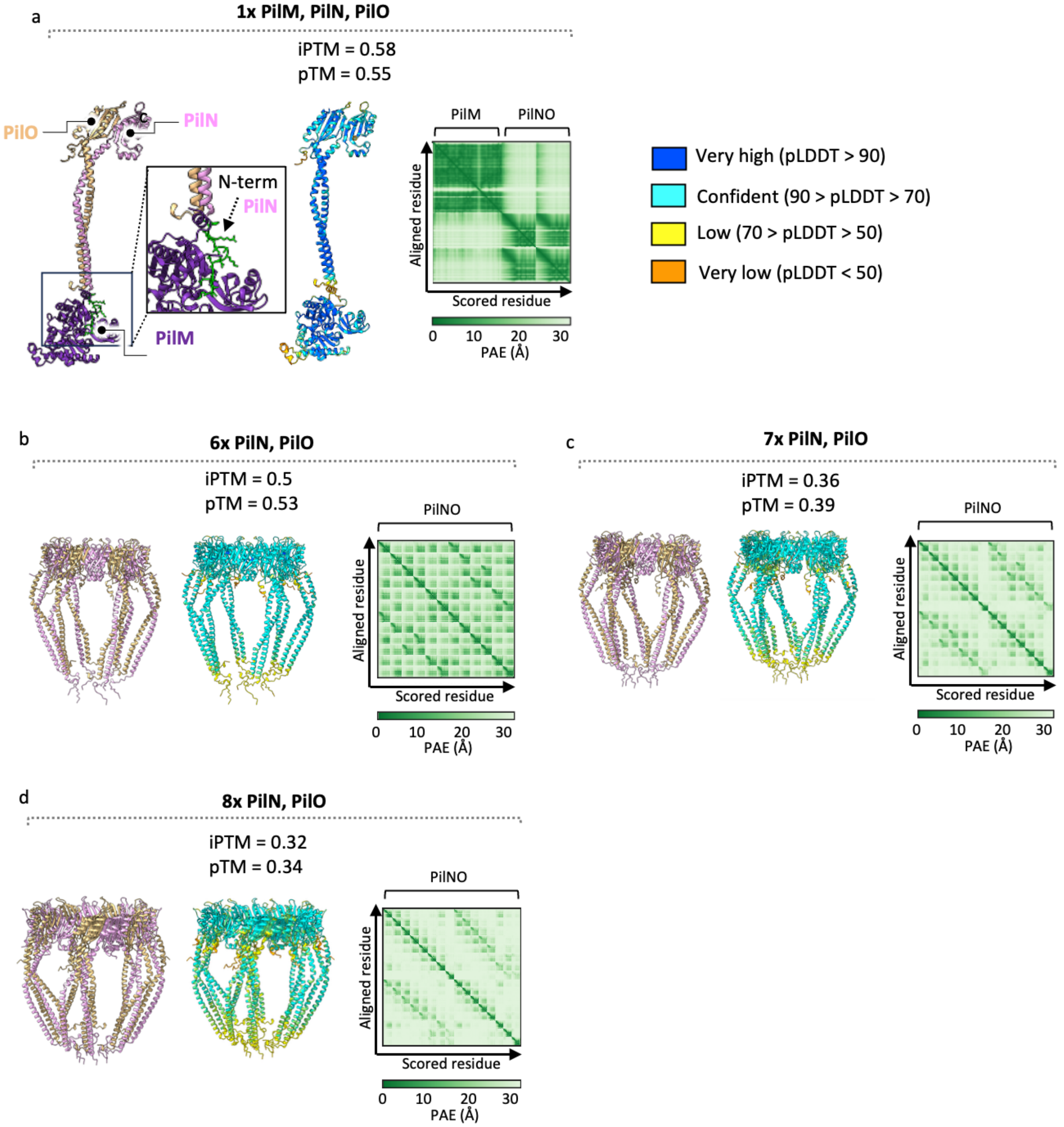

Supplementary Fig. 5. AlphaFold predictions of higher order stoichiometries of PilNO

(a) AlphaFold prediction for a complex of PilM, PilN and PilO, as per Supplementary Fig. 4a.

(b-d) Predictions without PilM, and with increasing numbers of PilN and PilO subunits. Per-residue pLDDT scores are shown according to the key (top right), with corresponding ipTM and pTM values and predicted alignment error (PAE).

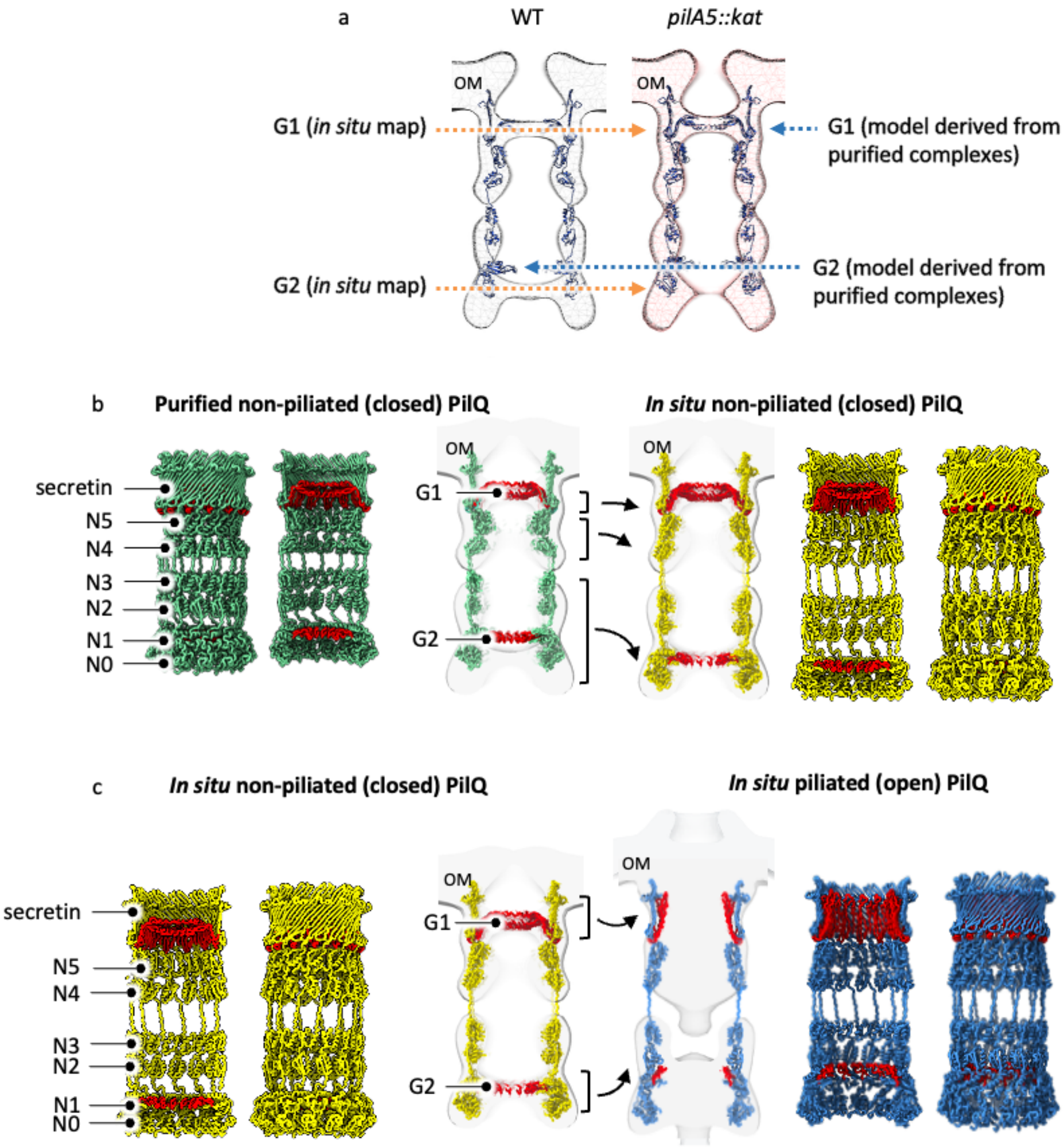

Supplementary Fig. 6. The structure of PilQ and gate opening

(a) A single-particle cryoEM map of purified *T. thermophilus* PilQ was used to generate a homology model, kindly provided by the original authors[5]. Left to right: sub-tomogram average maps of the WT (grey) and *pilA5::kat* mutant (orange) obtained *in situ*, overlaid with the PilQ homology model (dark blue ribbon). Sub-tomogram averages were collected with different microscopes at different centres (Supplementary Table 1), showing that PilQ length and the distance between the gates (blue arrowheads in model, orange *in situ*) decrease on purification. OM, outer membrane.

(b) The PilQ homology model (green, shown in full and sliced through the centre) was re-modelled (yellow, shown in full and sliced through the centre) to fit the *in situ* sub-tomogram average of the non-piliated (closed) state of PilQ (see Supplementary Note 3). This involved downward translation

of domains N0-N3, downward translation and tilting of domain N4, and outward tilting of domain N5. The gates of PilQ are indicated in red. OM, outer membrane.

(c) To model the open form of PilQ, Gates 1 and 2 were adjusted according to PDB 6Q15[28] (see Supplementary Note 3). Closed PilQ is shown in yellow, open PilQ in blue (both shown in full and sliced through the centre), with gates indicated in red. OM, outer membrane.

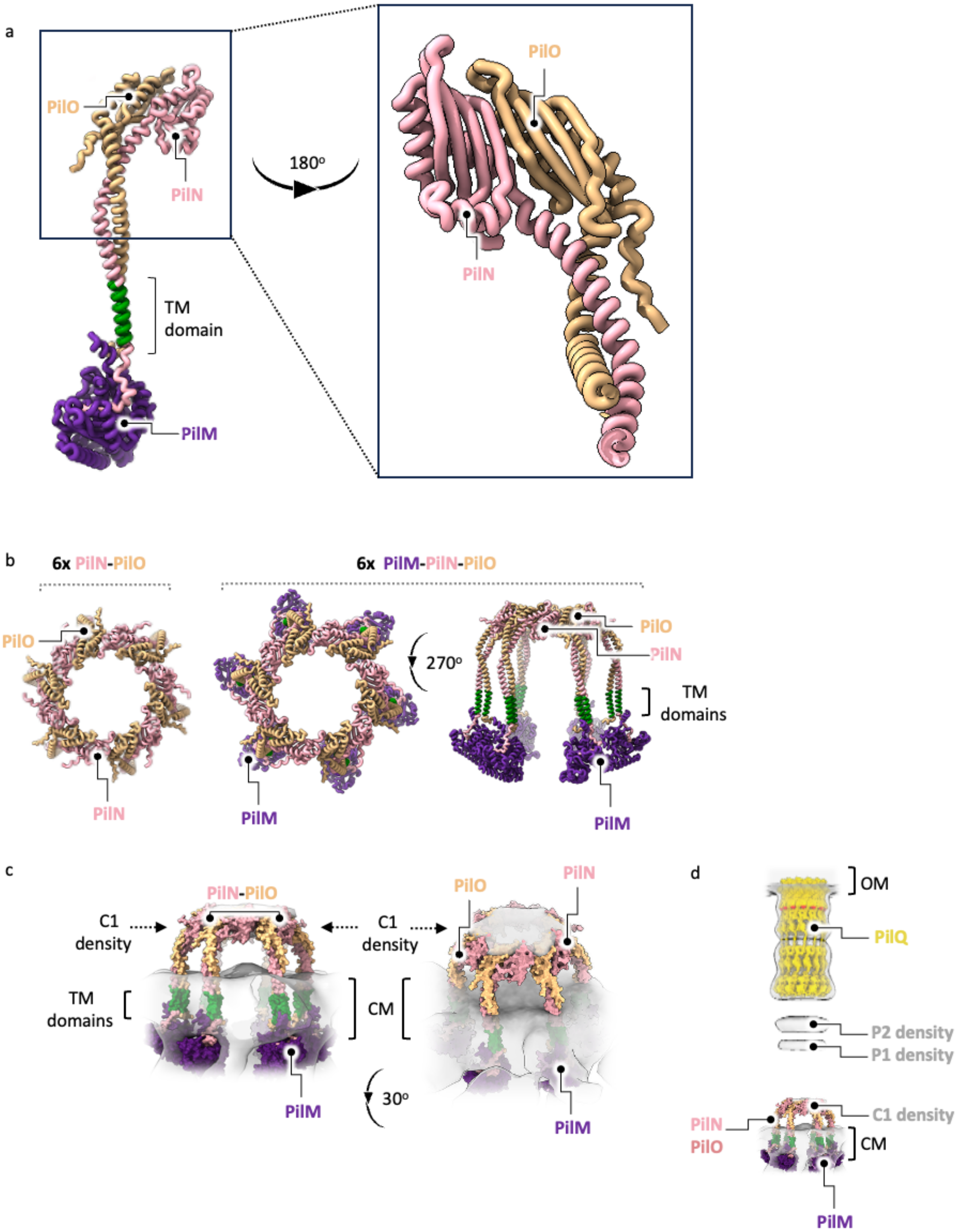

Supplementary Fig. 7. Predictions and fit of PilMNO into the sub-tomogram average map

(a) AlphaFold prediction for a complex of PilM, PilN and PilO (as in Supplementary Fig. 4a). The predicted transmembrane (TM) domain is indicated in green. A 180° rotated view shows the β-sheet domains of PilN and PilO, showing curvature in the prediction.

(b) A 6-fold complex of (left) PilNO and (right) PilMNO built from the AlphaFold prediction, with 5 additional copies arranged in a ring to form a heterohexamer. Ribbon representation views are shown towards the cytoplasmic membrane (CM) and in plane with the membrane; predicted transmembrane (TM) domains are indicated in green.

(c) The 6-fold PilMNO complex in surface representation, shown in side and 30° tilted views, placed into the map. The PilN and PilO transmembrane (TM) domains (green) align with the position of the CM.

(d) Sub-tomogram average of the T4P machinery showing the positions of PilQ and PilMNO placed into the map. OM, outer membrane; CM, cytoplasmic membrane.

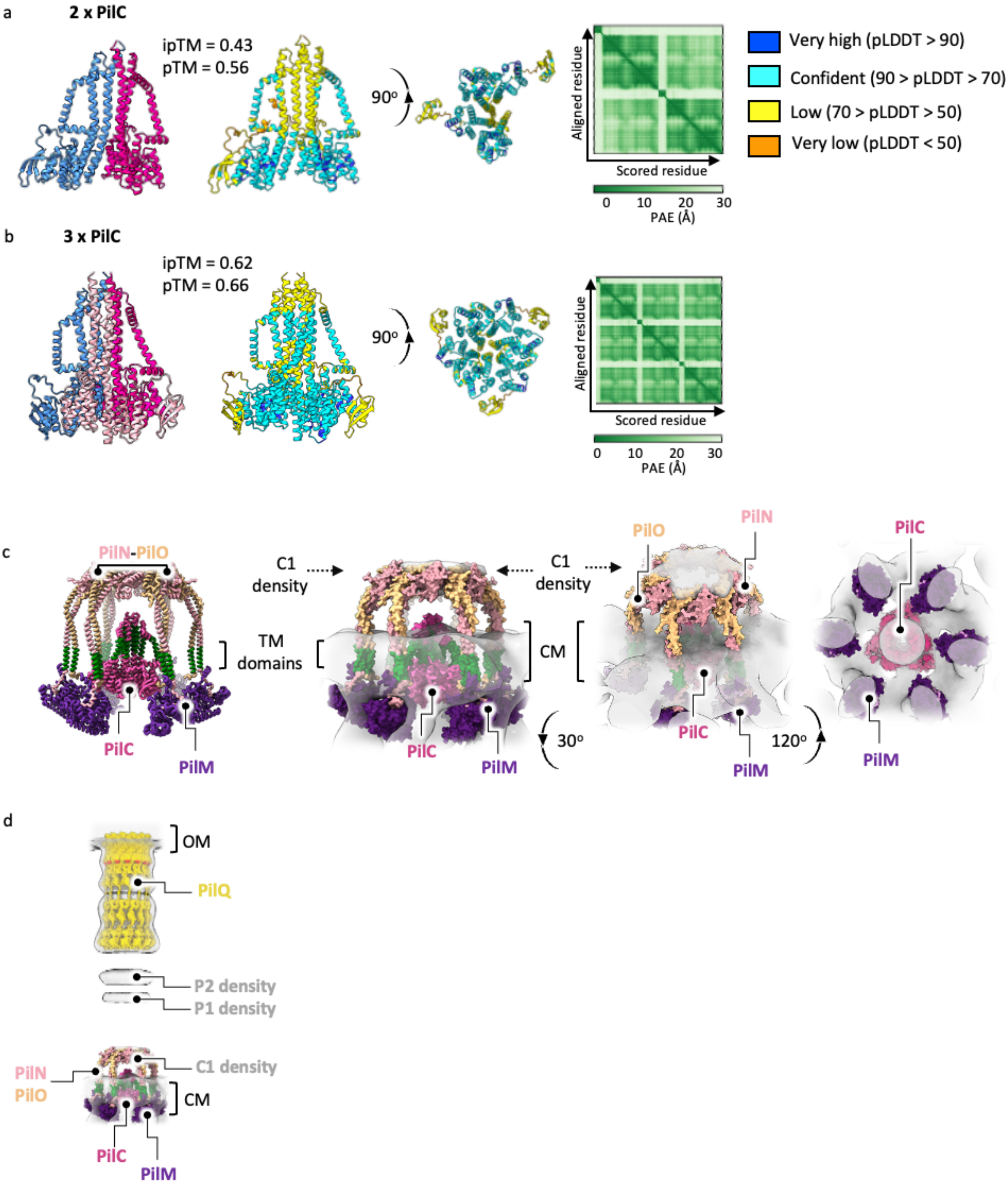

Supplementary Fig. 8. Predictions of PilC and fit into the sub-tomogram average map

(a, b) AlphaFold predictions for (a) a dimer and (b) trimer of PilC, shown in side view with each chain coloured differently in ribbon representation. The same views are shown rotated 90°, coloured by per-residue pLDDT score according to the key (top right), with corresponding ipTM and pTM values and predicted alignment error (PAE). The N-terminal 38 residues of PilC are disordered and excluded from the prediction.

(c) A 6-fold complex of PilMNO with a trimer of PilC shown from left to right: in-plane with the cytoplasmic membrane (CM) with predicted transmembrane (TM) domains in green in ribbon representation; a surface representation of the same complex placed into the sub-tomogram average map; a 30° tilted view; a subsequent 120° tilted view.

(d) Sub-tomogram average of the T4P machinery showing the positions of PilQ, PilM, PilN, PilO, and PilC placed into the sub-tomogram average map. OM, outer membrane; CM, cytoplasmic membrane.

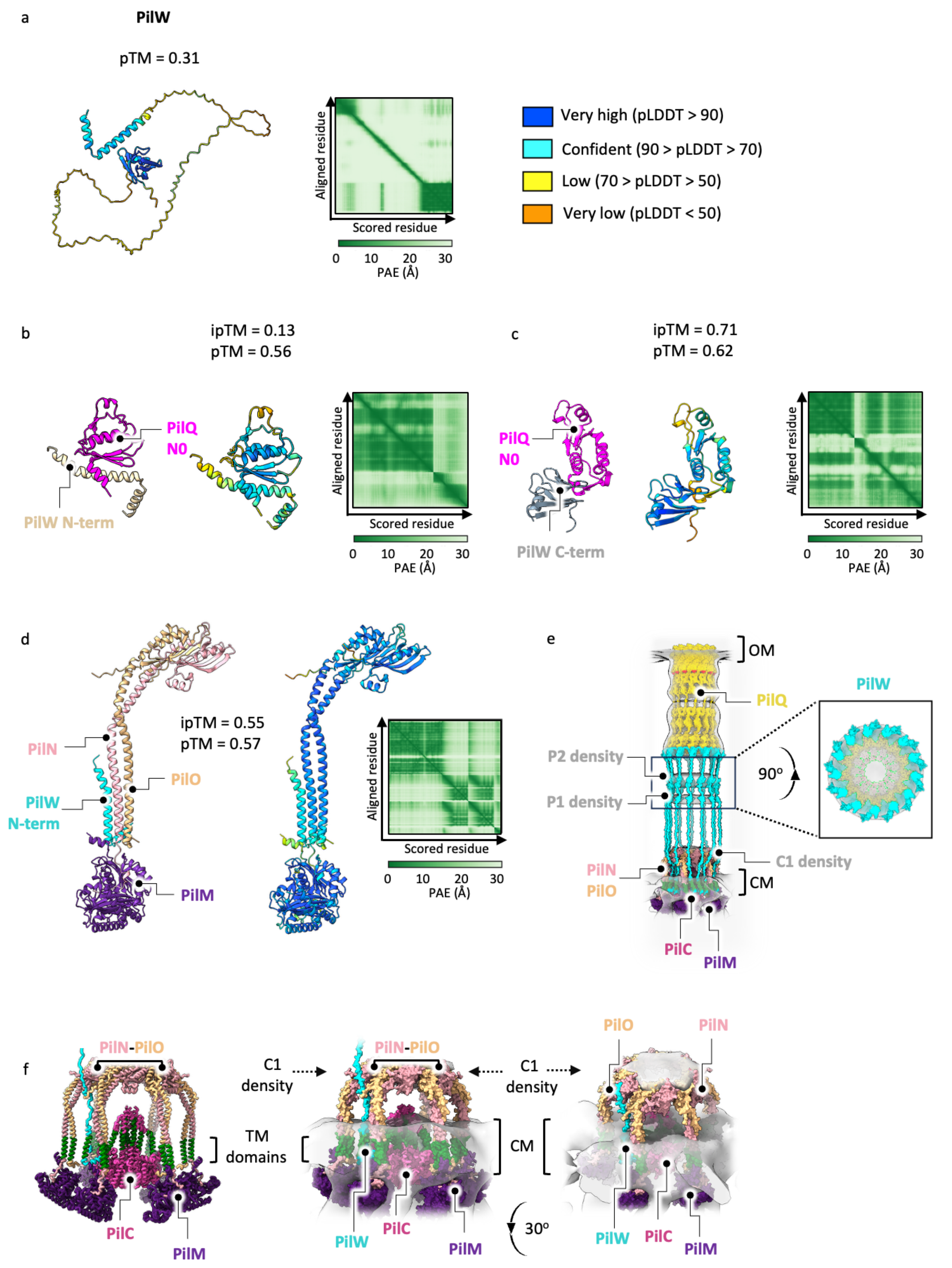

Supplementary Fig. 9. AlphaFold predictions of PilW

(a) AlphaFold prediction for PilW coloured by per-residue pLDDT score according to the key (top right), with corresponding pTM value and predicted alignment error (PAE).

(b) AlphaFold prediction of the N0 domain of PilQ (residues 1-222) in complex with the N-terminus of PilW (residues 1-50), with each protein coloured differently and by pLDDT score per residue, with corresponding PAE.

(c) AlphaFold prediction of the N0 domain of PilQ (residues 1-222) in complex with the C-terminus of PilW (residues 216-292) with each protein coloured differently and by pLDDT score per residue, with corresponding ipTM and pTM values and PAE.

(d) AlphaFold prediction for PilMNO in complex with the N-terminal α-helix of PilW (residues 1-50), with each protein coloured differently and by pLDDT score per residue, with corresponding ipTM and pTM values and PAE.

(e) Sub-tomogram average of the T4P machinery, showing positions of PilQ, PilM, PilN, PilO, PilC, and PilW, placed into the sub-tomogram average map. OM, outer membrane; CM, cytoplasmic membrane.

(f) A 6-fold complex of PilMNO and PilC with the N-terminus of PilW, shown from left to right: in-plane with the cytoplasmic membrane (CM) with predicted transmembrane (TM) domains green in ribbon representation, the same complex as a surface representation placed into the sub-tomogram average map; and a 30° tilted view.

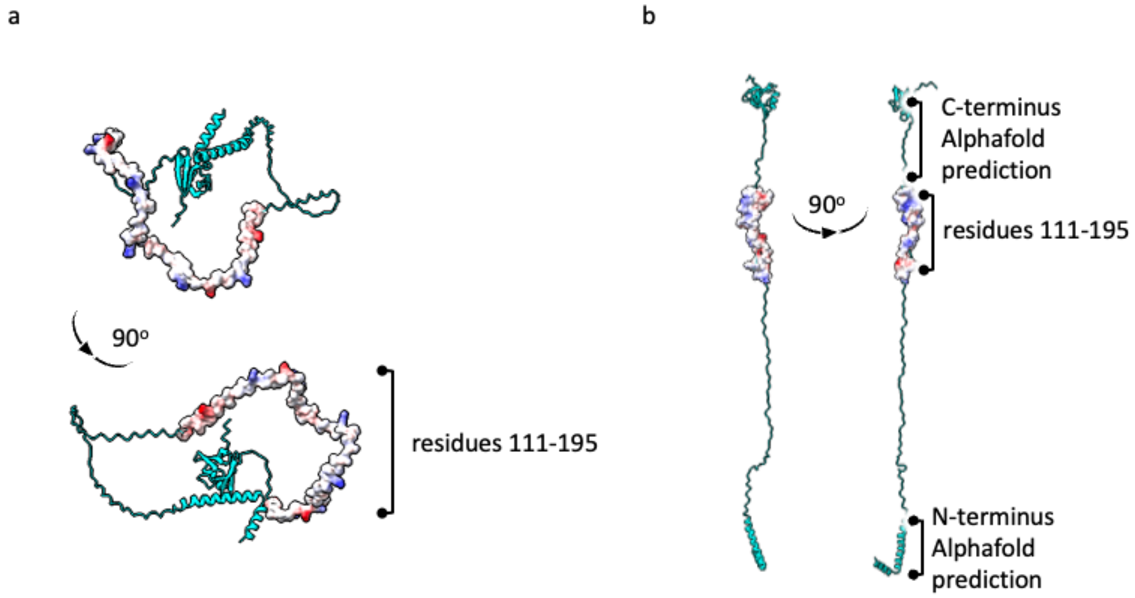

Supplementary Fig. 10. The PilW folded domain

(a) AlphaFold prediction of PilW, showing the folded domain (residues 111–195) with electrostatic surface potential highlighting regularly spaced opposing charges. The folded N- and C-termini are shown in teal ribbon representation.

(b) AlphaFold prediction of PilW N- and C-termini with a mostly unfolded linker domain, with residues 111-195 modelled in a more compact conformation coloured by electrostatic surface potential.

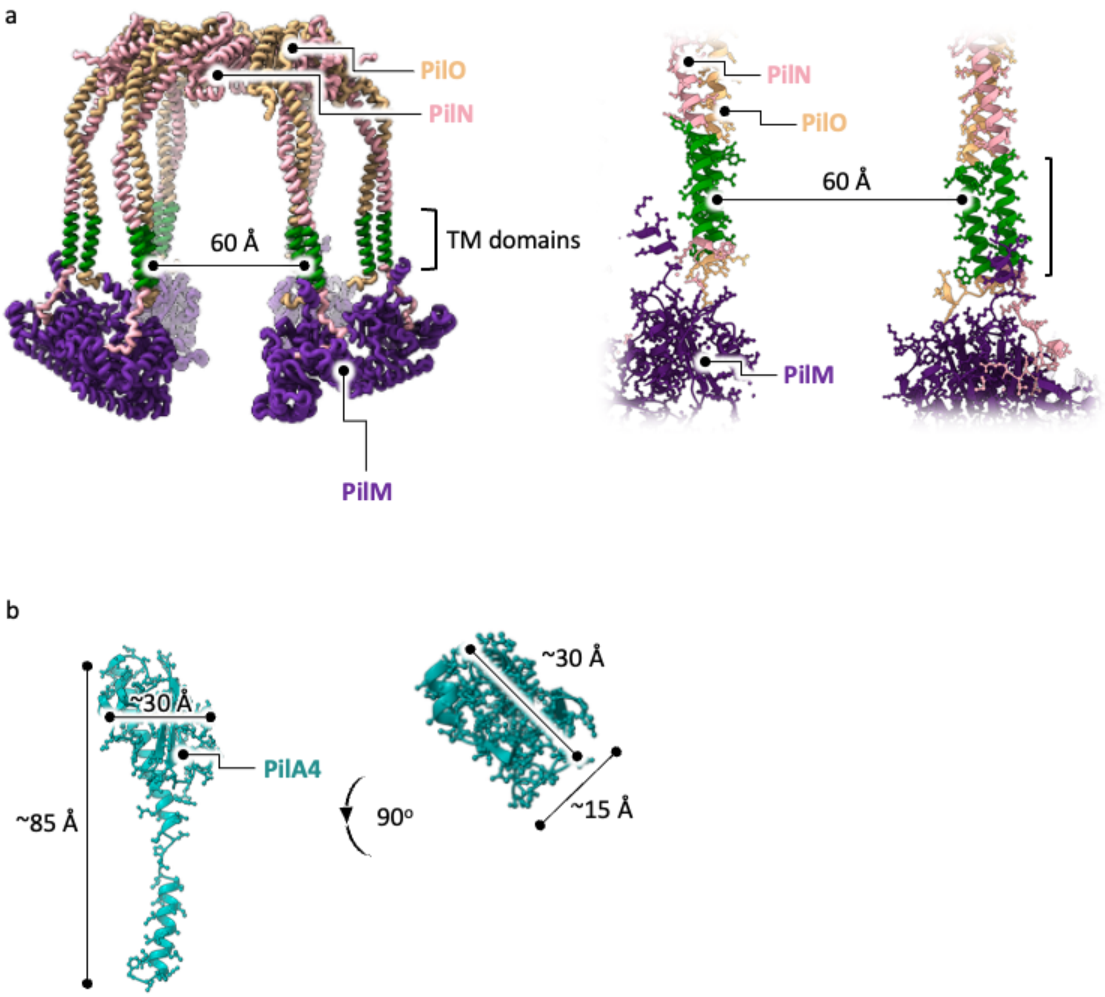

Supplementary Fig. 11. Distances between neighbouring PilMNO domains

(a) Left to right: model of PilMNO with each protein coloured differently and transmembrane (TM) domains indicated in ribbon representation; side-chains and distances between the α-helices of heterodimeric PilNO units are shown.

(b) Experimental structure of a PilA4 subunit with distances indicated.

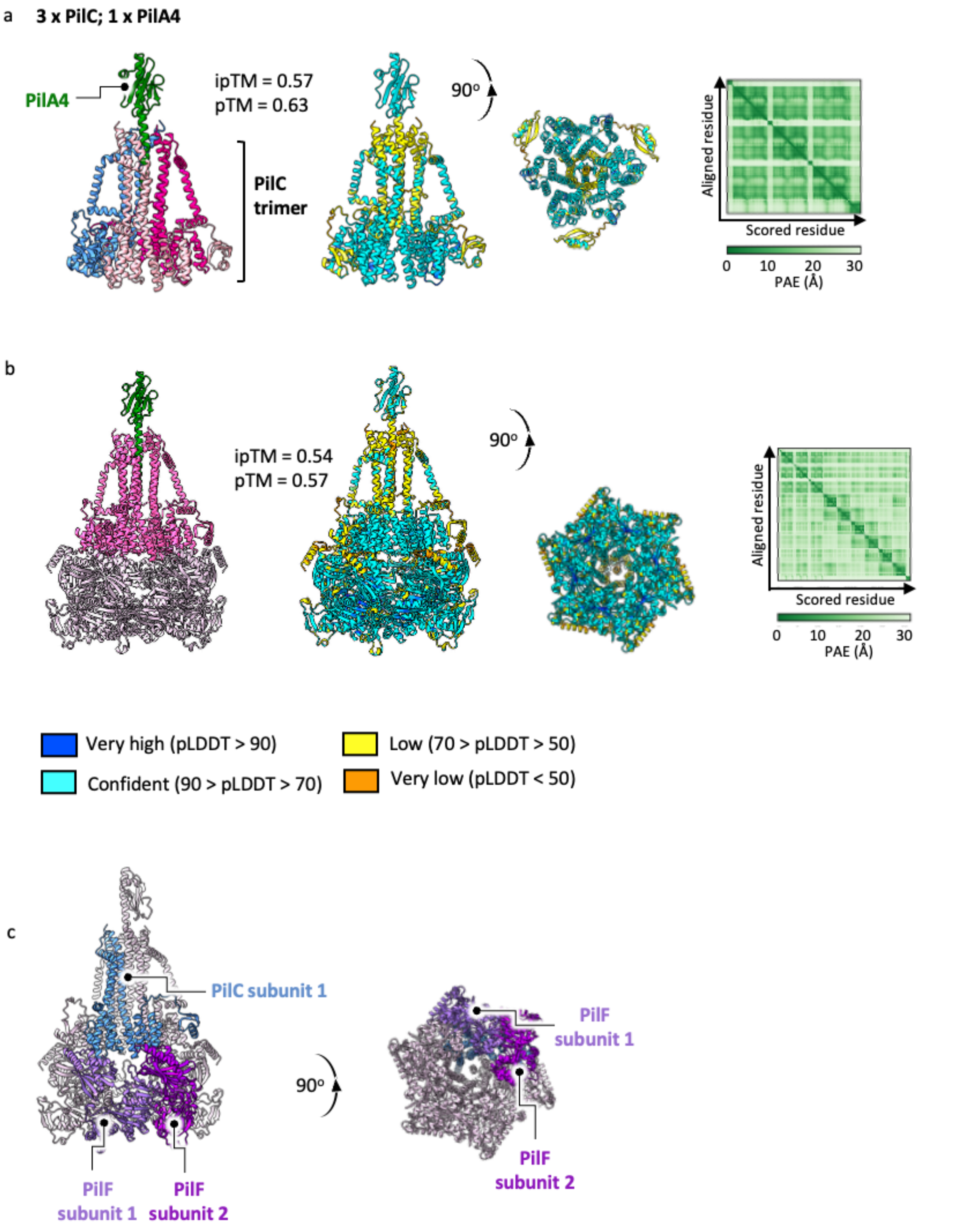

Supplementary Fig. 12. Predictions of PilC with PilA4 and PilF

(a) AlphaFold prediction of a trimer of PilC with a single PilA4 subunit, shown in side view with each protein coloured differently. The same view is coloured by per-residue pLDDT score according to the key (below (b)), with corresponding ipTM and pTM values and predicted alignment error (PAE). The

N-terminal 38 residues of PilC are disordered and excluded, as their inclusion substantially reduced confidence scores.

(b) AlphaFold prediction of a PilC trimer including a hexamer of the C-terminal ATPase domain (residues 477–889) of PilF, shown in side view with each protein coloured differently with metrics as in (a).

(c) Alignment of PilC subunit 1 with PilF subunits 1 and 2.

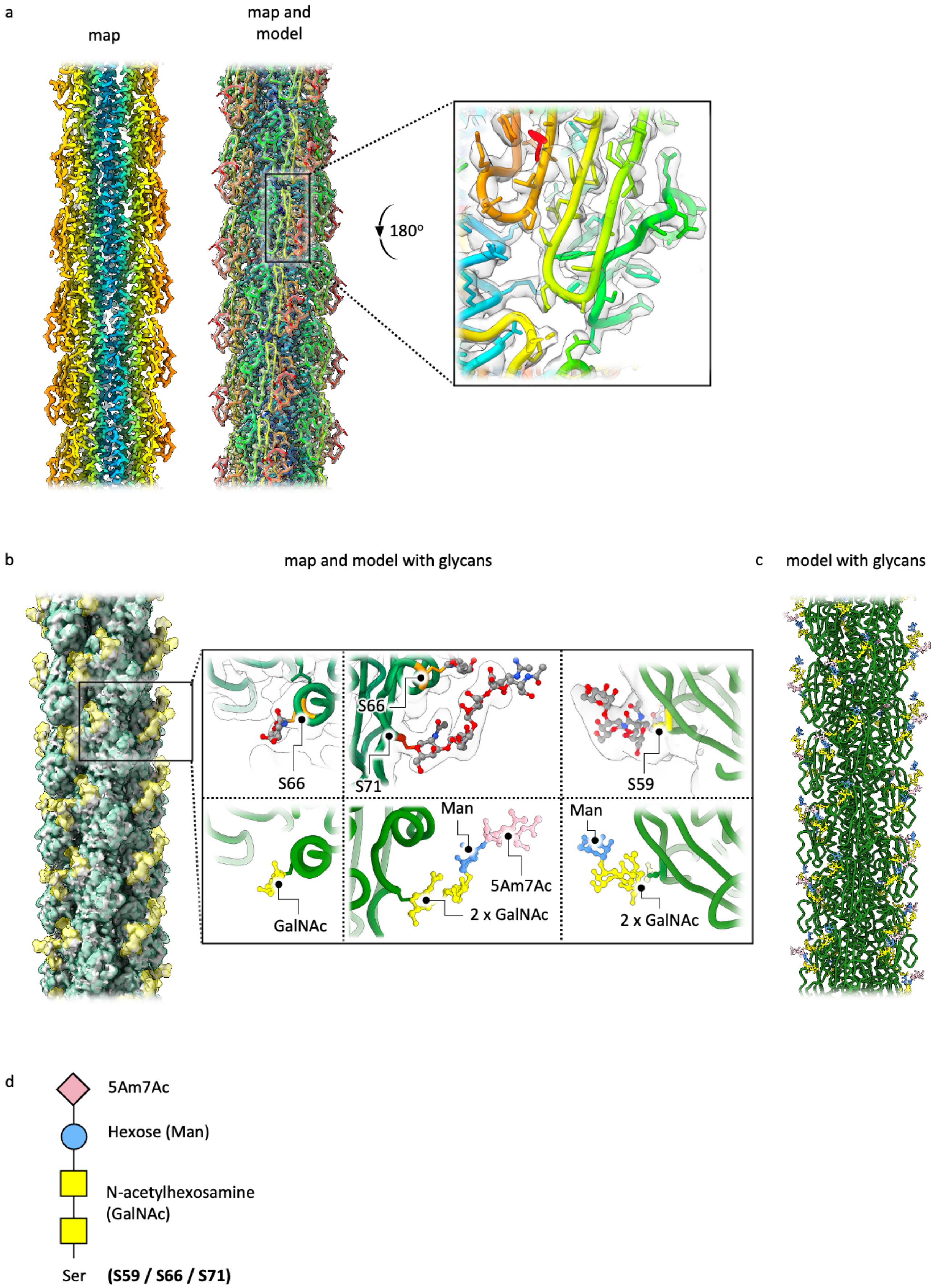

Supplementary Fig. 13. The wide T4P structure

(a) Left: cryoEM map of the wide (PilA4) T4P. Right: model built into the map, coloured in rainbow representation, with an boxed enlarged view showing the fit of the model to the map.

(b) Wide T4P model in surface representation with glycosylation sites indicated in yellow, overlaid on the cryoEM map (grey). Boxed enlarged views show cryoEM map density on serine residues (S59, S66 and S71) unaccounted for by the protein chain, where glycans were built in. The full-length sugar (5Am7Ac-α(1-4)Man-α(1-3)GalNAc-α(1,3)GalNAc-α-Ser) was built at S71, with truncated versions at S59 and S66.

(c) Wide T4P model shown with glycans coloured according to the key in (d).

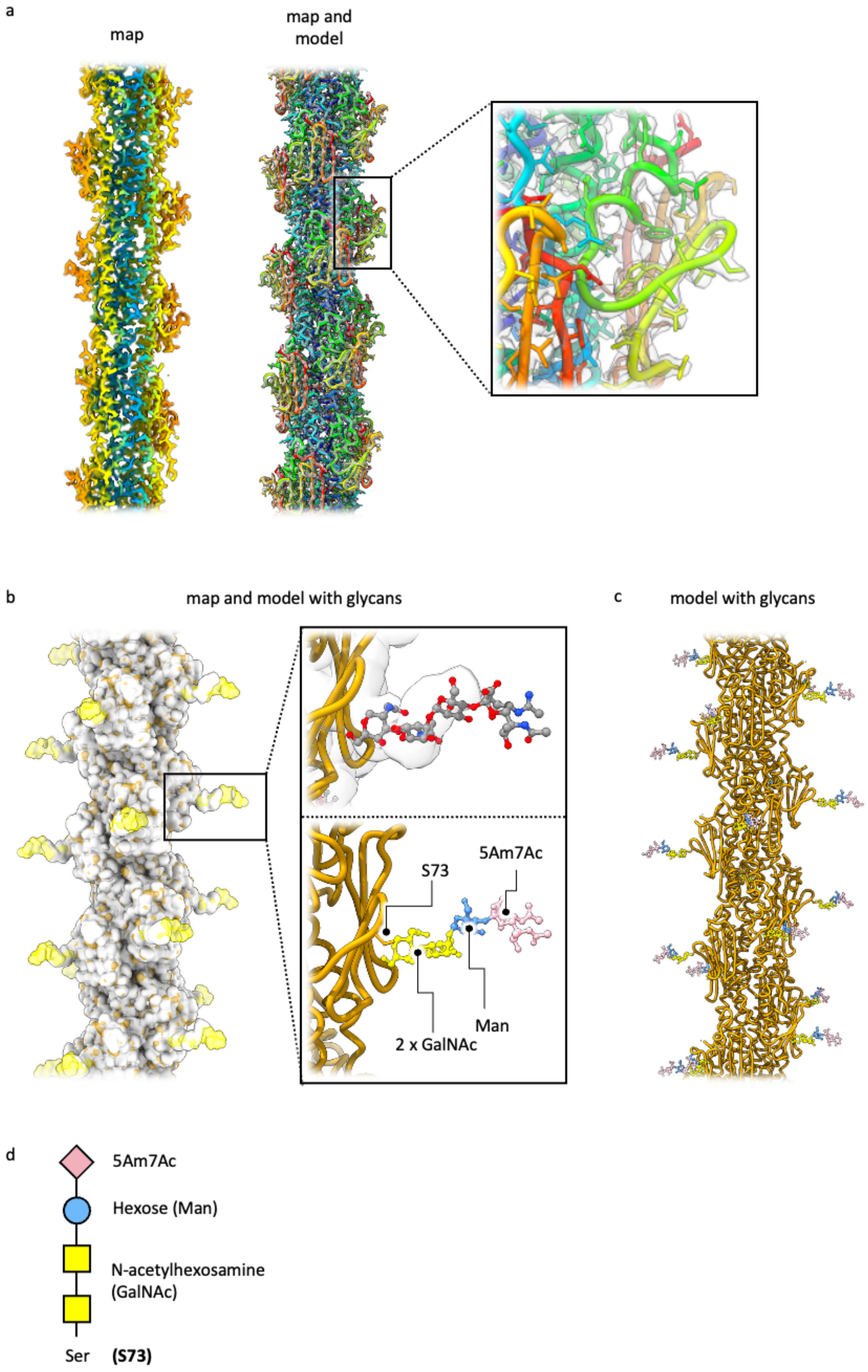

Supplementary Fig. 14. The narrow T4P structure

(a) Left: cryoEM map of the narrow (PilA5) T4P. Right: model built into the map, coloured in rainbow representation, with an boxed enlarged view showing the fit of the model to the map.

(b) Narrow T4P model with glycosylation sites indicated in yellow, overlaid on the cryoEM map (grey). Boxed enlarged views show cryoEM map density on serine residue S73 unaccounted for by the protein chain, where the full-length sugar (5Am7Ac-α(1-4)Man-α(1-3)GalNAc-α(1,3)GalNAc-α-Ser) was built.

(c) Narrow T4P model shown with glycans coloured according to the key in (d).

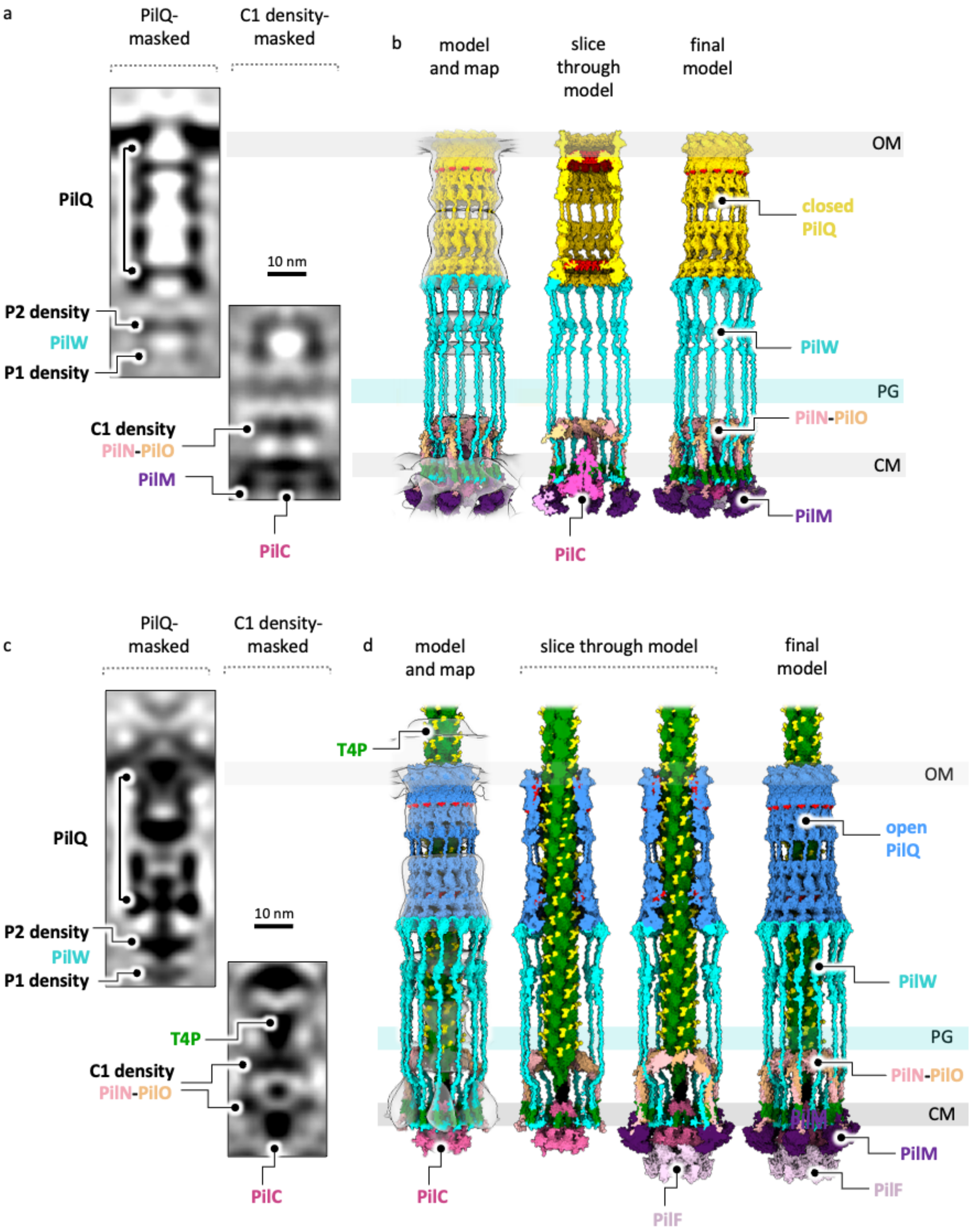

Supplementary Fig. 15. Docking structures into sub-tomogram averages of the T4P machinery in non-piliated and piliated states

(a) Non-piliated PilQ-masked and C1 density-masked sub-tomogram average maps, overlaid at the P1/P2 density, with all densities and corresponding protein names indicated. The text colours of the protein names match the colours of the corresponding structures in (b). OM, outer membrane; PG, peptidoglycan; CM, cytoplasmic membrane.

(b) Hypothetical models docked into the non-piliated state sub-tomogram average maps, from left to right: complete model in the maps, model alone without the maps, a central slice of the model revealing the gates in PilQ (red), and a full view without the maps.

(c) Piliated PilQ-masked and C1 density-masked sub-tomogram average maps, overlaid at the P1/P2 density with all densities and corresponding protein names indicated.

(d) Hypothetical models docked into the piliated state sub-tomogram average maps, from left to right: complete model in the maps; model alone without the maps, a central slice of the model without and with PilF; and a full view without the maps.

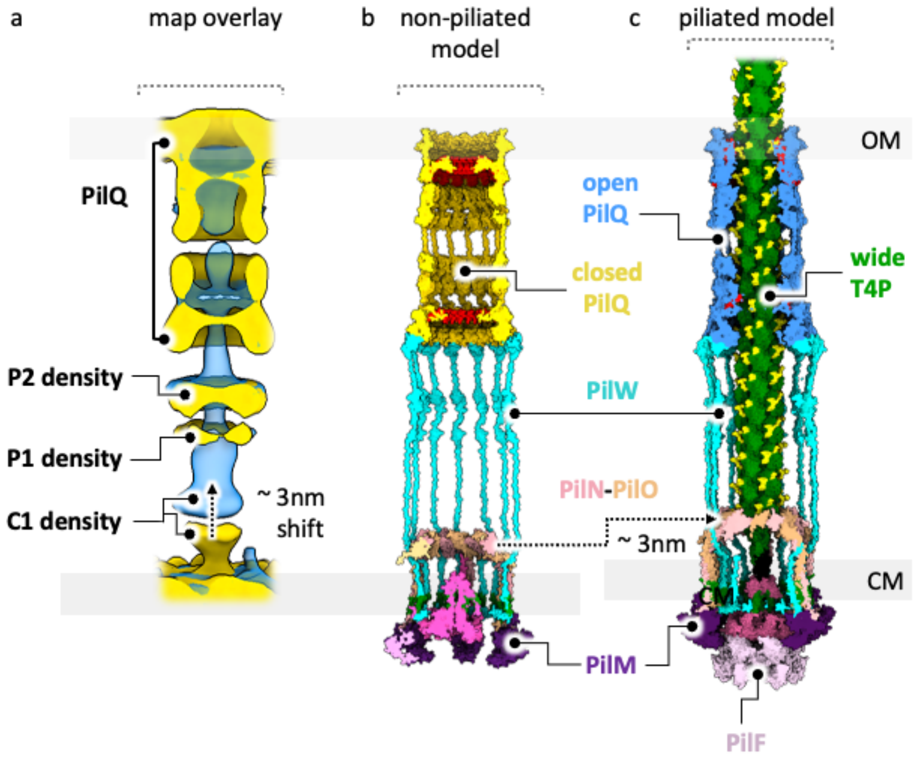

Supplementary Fig. 16. Comparisons of non-piliated and piliated forms of the machinery

(a) Overlay of sub-tomogram average maps of non-piliated (yellow) and piliated (blue) forms of the T4P machinery, calculated using C13 symmetry to provide consistent alignment via the P1/P2 densities.

(b, c) Hypothetical models of the T4P machinery in (b) non-piliated and (c) piliated forms, sliced through the centre. The non-piliated form is shown with the wide T4P docked into PilQ with glycans in yellow. The PilF structure is PDB 6F8L[9]. Dashed arrows indicate an upward shift of the cytoplasmic membrane assembly platform by ~3 nm. OM, outer membrane; CM, cytoplasmic membrane.

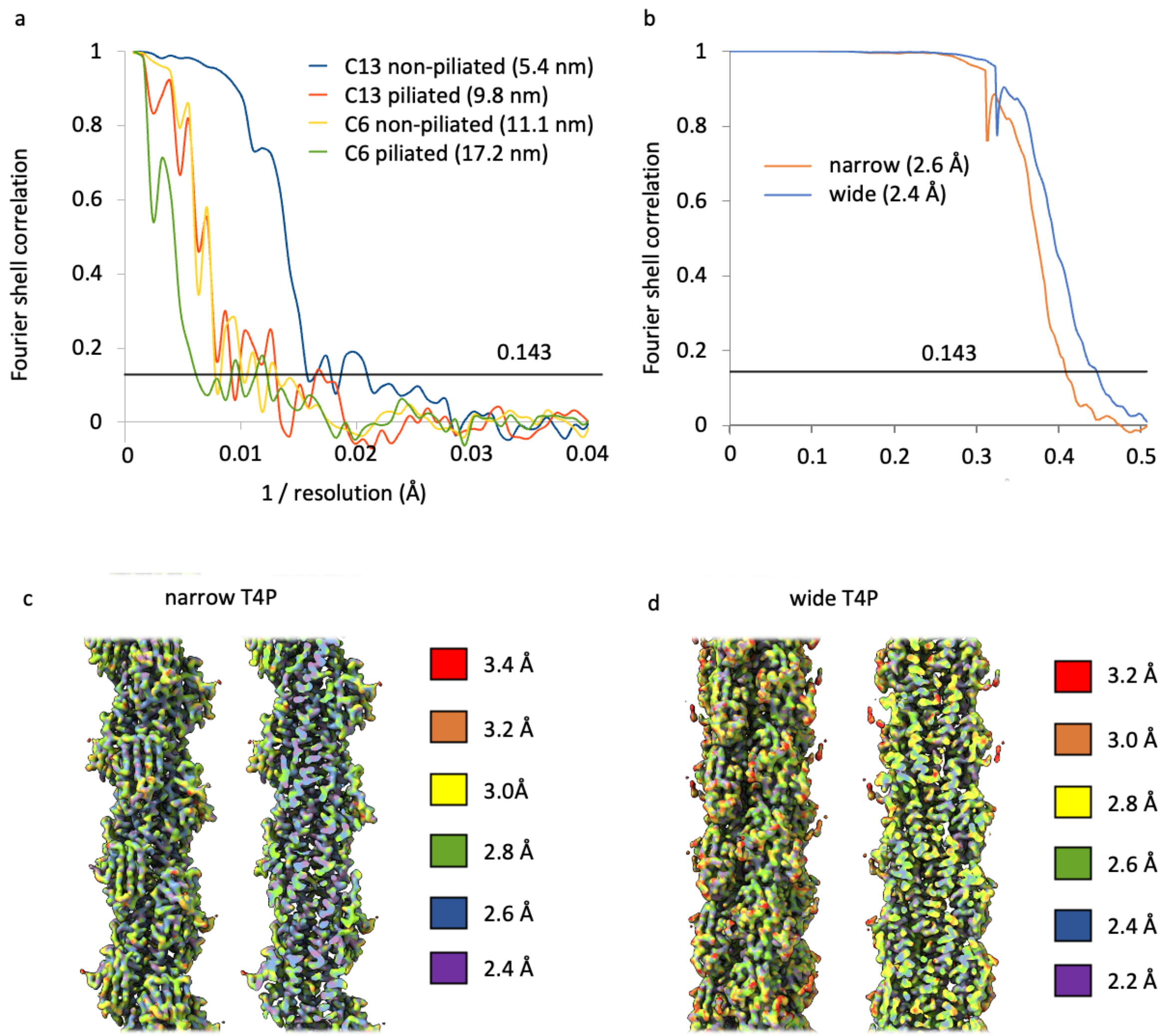

Supplementary Fig. 17. Resolution estimates

(a, b) FSC curves for (a) sub-tomogram averages used to build the T4P machinery model and (b) for single-particle T4P structures.

(c, d) Local resolution estimates for the (c) narrow (PilA5) T4P and (d) wide (PilA4) T4P. For each, a surface view is shown on the left and a slice through the centre on the right.

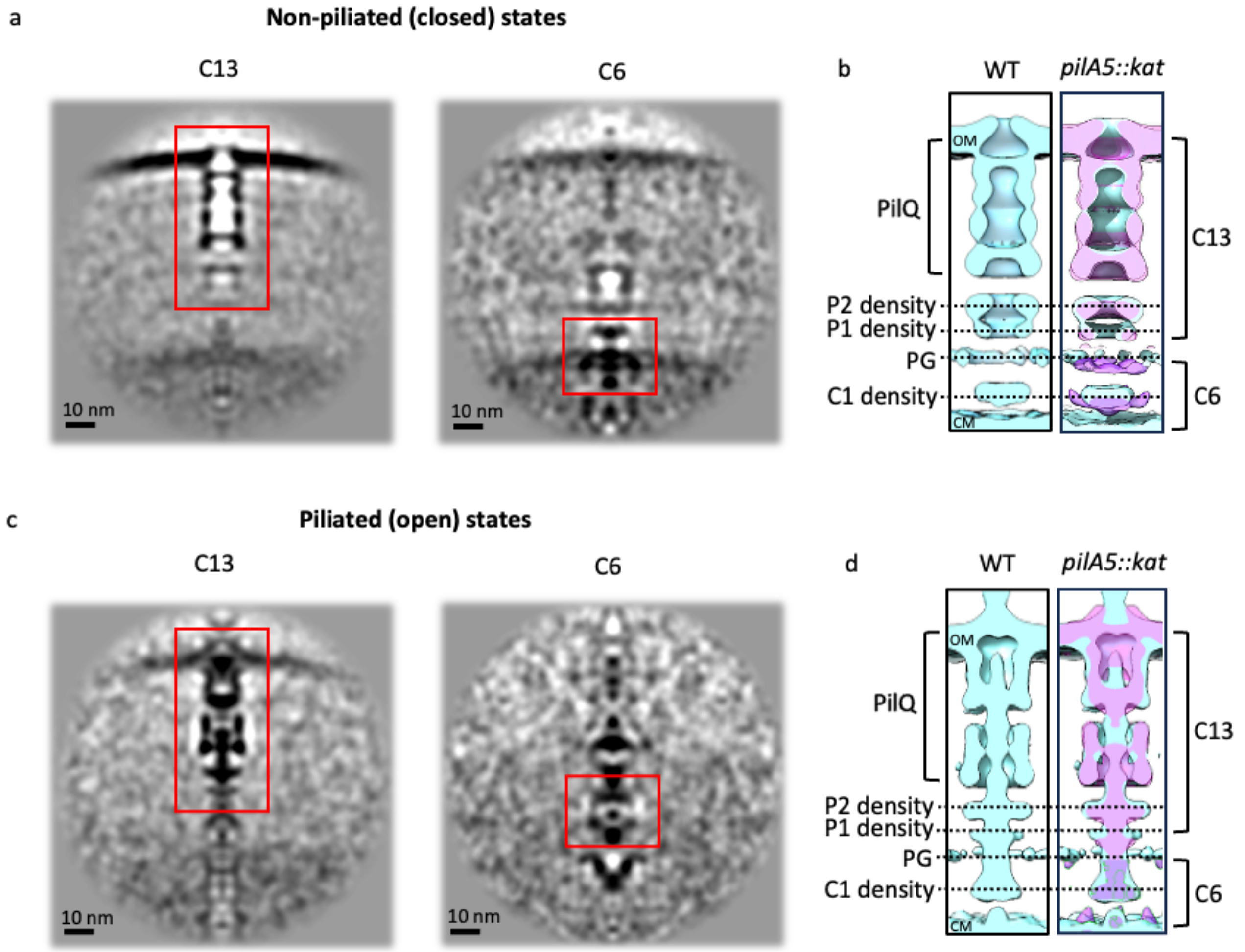

Supplementary Fig. 18. Sub-tomogram averages used to build the model

Slices through sub-tomogram averages obtained from the wide pilus only expressing mutant (*pilA5::kat*) in (a) non-piliated (closed) and (c) piliated (open) states. C13 (PilQ-masked) and C6 (C1 density-masked) sub-tomogram average maps are shown. Red boxed regions indicate regions used to build the model. Corresponding panels in (b) and (d) show the volumes (purple) displayed alongside and overlaid with the WT data (expressing both wide and narrow T4P)[31] to show their similarity. OM, outer membrane; CM, cytoplasmic membrane; PG, peptidoglycan.

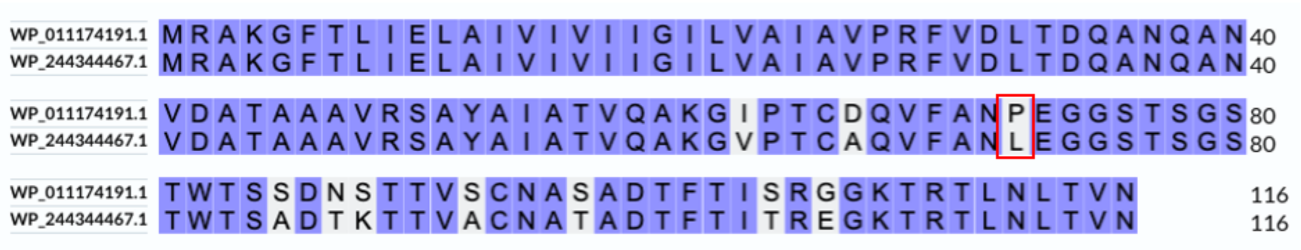

Supplementary Fig. 19. PilA5 sequence alignment.

A selection of *T. thermophilus* pilin sequences with similarity to PilA5 were identified and aligned using Uniprot. The two examples shown highlight sequence variation, with the boxed region indicating that either proline or leucine can occupy the same position.

Strain	Microscope	Number of tomograms	Non-piliated (PilQ closed) state		Piliated (PilQ open) state	
			T4P complexes in average	Resolution estimate (nm)	T4P complexes in average	Resolution estimate (nm)
WT	Krios	unknown* (~7)	#332	4	#26	5
pilM::kat	Talos	26	49	11	n/a	n/a
pilN::kat	Krios	55	57	6	n/a	n/a
pilO::kat	Krios	80	41	5	n/a	n/a
pilWΔ163-216	Krios	113	100	6	9	30

Supplementary Table 1. Data collection for sub-tomogram averages of mutants used to identify proteins in the hypothetical model

Sub-tomogram averaging parameters include strain, microscope type, number of tomograms collected, number of T4P complexes used in the average, and resolution estimates reported at the 0.5 Fourier Shell Correlation (FSC) criterion. *We also report the parameters from the WT data collected in our previous work (EMD-3021 and EMD-3023). The number of tomograms from which particles were extracted from the WT is unknown, as they were processed elsewhere several years prior to this work[31]. Since that earlier work, advances in data processing have changed how symmetry is handled between the two studies. #Previously, symmetry could only be accounted for by manual expansion. In contrast, the current study employs automated symmetry expansion, splitting particles into half-sets, and performing separate refinement for different symmetry classes. Despite differences in particle numbers and the use of different microscopes for data collection, the impact on resolution estimates in the nanometre range are likely negligible.

Supplementary References

1. Karuppiah, V. & Derrick, J. P. Structure of the PilM-PilN inner membrane type IV pilus biogenesis complex from *Thermus thermophilus*. *J Biol Chem* **286**, 24434 (2011).
2. Sampaleanu, L. M. *et al.* Periplasmic domains of *Pseudomonas aeruginosa* PilN and PilO form a stable heterodimeric complex. *J Mol Biol* **394**, 143–159 (2009).
3. Abramson, J. *et al.* Accurate structure prediction of biomolecular interactions with AlphaFold3. **630**, 493–500 (2024).
4. Pettersen, E. F. *et al.* UCSF Chimera--a visualization system for exploratory research and analysis. *J Comput Chem* **25**, 1605–1612 (2004).
5. D'Imprima, E. *et al.* Cryo-EM structure of the bifunctional secretin complex of *Thermus thermophilus*. *Elife* **6:** e30483, (2017).
6. Emsley, P., Lohkamp, B., Scott, W. G. & Cowtan, K. Features and development of Coot. *Acta Crystallogr D Biol Crystallogr* **66**, 486–501 (2010).
7. Murshudov, G. N.; Vagin, A. A. & Dodson, E. J. Refinement of macromolecular structures by the maximum-likelihood method. *Acta Crystallogr D Biol Crystallogr* **53**, 240-255 (1997).
8. Karuppiah, V., Collins, R. F., Thistlethwaite, A., Gao, Y. & Derrick, J. P. Structure and assembly of an inner membrane platform for initiation of type IV pilus biogenesis. *Proc Natl Acad Sci U S A* **110**, E4638–E4647 (2013).
9. Collins, R. *et al.* Structural cycle of the *Thermus thermophilus* PilF ATPase: the powering of type IVa pilus assembly. *Sci Rep* **8**, 1–13 (2018).
10. Hallgren, J. *et al.* DeepTMHMM predicts alpha and beta transmembrane proteins using deep neural networks. *bioRxiv* 2022.04.08.487609 (2022) doi:10.1101/2022.04.08.487609.
11. Chang, Y. W. *et al.* Architecture of the type IVa pilus machine. *Science* **351**, (2016).
12. Chang, Y. W. *et al.* Architecture of the *Vibrio cholerae* toxin-coregulated pilus machine revealed by electron cryotomography. *Nature Microbiology* **2**, 1–7 (2017).
13. Guo, S. *et al*. PilY1 regulates the dynamic architecture of the type IV pilus machine in *Pseudomonas aeruginosa*. *Nat Commun* **15:1** 15, 1–12 (2024).
14. Karuppiah, V., Hassan, D., Saleem, M. & Derrick, J. P. Structure and oligomerization of the PilC type IV pilus biogenesis protein from *Thermus thermophilus*. *Proteins: Structure, Function, and Bioinformatics* **78**, 2049–2057 (2010).

15. Bischof, L. F., Friedrich, C., Harms, A., Søgaard-Andersen, L. & Van Der Does, C. The Type IV pilus assembly ATPase PilB of *Myxococcus xanthus* interacts with the inner membrane platform protein PilC and the nucleotide-binding protein PilM. *J Biol Chem* **291**, 6946–6957 (2016).
16. Abendroth, J. *et al.* The three-dimensional structure of the cytoplasmic domains of EpsF from the type 2 secretion system of *Vibrio cholerae*. *J Struct Biol* **166**, 303–315 (2009).
17. Takhar, H. K., Kemp, K., Kim, M., Howell, P. L. & Burrows, L. L. The platform protein is essential for type IV pilus biogenesis. *Journal of Biological Chemistry* **288**, 9721–9728 (2013).
18. Guilvout, I. *et al.* Membrane platform protein PulF of the *Klebsiella* type II secretion system forms a trimeric ion channel essential for endopilus assembly and protein secretion. *mBio* **15**, (2024).
19. Pelicic, V. Mechanism of assembly of type 4 filaments: everything you always wanted to know (but were afraid to ask). *Microbiology* **169**, 001311 (2023).
20. Rumszauer, J., Schwarzenlander, C. & Averhoff, B. Identification, subcellular localization and functional interactions of PilMNOWQ and PilA4 involved in transformation competency and pilus biogenesis in the thermophilic bacterium *Thermus thermophilus* HB27. *FEBS J* **273**, 3261–3272 (2006).
21. Yaman, D. & Averhoff, B. Identification of subcomplexes and protein-protein interactions in the DNA transporter of *Thermus thermophilus*. *BBA - Biomembranes* **1866,** 184363 (2024).
22. Li, C., Wallace, R. A., Black, W. P., Li, Y. & Yang, Z. Type IV pilus proteins form an integrated structure extending from the cytoplasm to the outer membrane. *PLoS One* **8**, (2013).
23. Tammam, S. *et al*. PilMNOPQ from the *Pseudomonas aeruginosa* type IV pilus system form a transenvelope protein interaction network that interacts with PilA. *J Bacteriol* **195**, 2126 (2013).
24. Bianchi, G., Longhi, S., Grandori, R. & Brocca, S. Relevance of electrostatic charges in compactness, aggregation, and phase separation of intrinsically disordered proteins. *Int J Mol Sci* **21**, 6208 (2020).
25. Balasingham, S. V. *et al*. Interactions between the lipoprotein PilP and the secretin PilQ in *Neisseria meningitidis. J Bacteriol* **189**, 5716–5727 (2007).
26. Berry, J. L. *et al*. Structure and assembly of a trans-periplasmic channel for type IV pili in *Neisseria meningitidis*. *PLoS Pathog* **8**, e1002923 (2012).
27. Conners, R. *et al.* CryoEM structure of the outer membrane secretin channel pIV from the f1 filamentous bacteriophage. *Nat Commun* **12**, 6316 (2021).
28. Hu, J. *et al.* T3S injectisome needle complex structures in four distinct states reveal the basis of membrane coupling and assembly. *Nature Microbiology* **4**, 2010–2019 (2019).

29. McCallum, M., Tammam, S., Khan, A., Burrows, L. L. & Lynne Howell, P. The molecular mechanism of the type IVa pilus motors. *Nat Commun* **8**, 1–10 (2017).
30. Neuhaus, A., Muniyandi, S., Salzer, R., Langer, J. D., Kruse, K., Sanders, K., Daum, B., Averhoff, B. & Gold, V. A. M. Cryo-electron microscopy reveals two distinct type IV pili assembled by the same bacterium. *Nat Commun* **11**, (2020).
31. Gold, V. A. M., Salzer, R., Averhoff, B. & Kühlbrandt, W. Structure of a type IV pilus machinery in the open and closed state. *Elife* **4**, (2015).
