# Supplementary material for: Structural insights into the Thermus thermophilus type IV pilus machinery assembling two distinct pili
Source: Commun Biol. 2026 Mar 31;9:474. doi: 10.1038/s42003-026-09762-0 (PMC13039148; doi:10.1038/s42003-026-09762-0)
Supplement: Supplementary file 2 — Description of Additional Supplementary Files [file 42003_2026_9762_MOESM2_ESM.pdf]

Description of Additional Supplementary File

File name: Supplementary Data 1

Description: The source data behind the graphs in Fig. 3 and Supplementary Fig. 3.
